# A Dual-Template Molecularly Imprinted Polymer to Inhibit Quorum Sensing Molecules: Theoretical Design, Optimized Synthesis, Physicochemical Characterization and Preliminary Microbiological Analysis

**DOI:** 10.3390/ijms26168015

**Published:** 2025-08-19

**Authors:** Khonzisizwe Somandi, Tama S. Mwale, Monika Sobiech, Dorota Klejn, Gillian D. Mahumane, Joanna Giebułtowicz, Sandy van Vuuren, Yahya E. Choonara, Piotr Luliński

**Affiliations:** 1 Wits Advanced Drug Delivery Platform Research Unit, 7 York Road, Parktown, Johannesburg 2193, South Africa; khonzisizwe.somandi@wits.ac.za (K.S.); 2179360@students.wits.ac.za (T.S.M.); gillian.mahumane@wits.ac.za (G.D.M.); 2 Department of Pharmacy and Pharmacology, School of Therapeutic Science, Faculty of Health Sciences, University of the Witwatersrand, 7 York Road, Parktown, Johannesburg 2193, South Africa; 3 Department of Organic and Physical Chemistry, Faculty of Pharmacy, Medical University of Warsaw, Banacha 1, 02-097 Warsaw, Poland; monika.sobiech@wum.edu.pl (M.S.); dorota.klejn@wum.edu.pl (D.K.); 4 Department of Drug Chemistry, Pharmaceutical and Biomedical Analysis Faculty of Pharmacy, Medical University of Warsaw, Banacha 1, 02-097 Warsaw, Poland; joanna.giebultowicz@wum.edu.pl; 5 Department of Pharmacy and Pharmacology, Faculty of Health Sciences, University of the Witwatersrand, 7 York Road, Parktown, Johannesburg 2193, South Africa; sandy.vanvuuren@wits.ac.za

**Keywords:** molecularly imprinted polymer, theoretical design, molecular docking, dual template strategy, autoinducer, anti-quorum sensing, violacein suppression, autoinducer-2 analog

## Abstract

Molecularly imprinted polymers (MIPs) have emerged as promising materials for selectively targeting biomolecules, including quorum sensing autoinducers that regulate bacterial communication and biofilm formation. In this study, both single-template and dual-template strategies were employed to design and synthesize MIPs capable of capturing autoinducer-2 analogs using (3R,4S)-tetrahydro-3,4-furandiol (**T1**) or (R/S) 2,2-dimethyl-1,3-dioxolane-4-methanol (**T2**) as the templates. This approach offers translational potential of a complementary or non-antibiotic strategy to conventional antimicrobial therapies in mitigating biofilm-associated infections. Computational modeling guided the rational selection of functional monomers, predicting favorable interaction energies (*ΔE_C_* up to −135 kcal·mol^−1^) and optimal hydrogen-bonding patterns to enhance template–polymer affinity. The synthesized MIPs were characterized using spectroscopic and microscopic techniques to confirm imprinting efficiency and structural integrity. The adsorption capacity measurements demonstrated higher adsorption capacity and selectivity of MIPs compared to non-imprinted polymers, with the highest selectivity equal to 3.36 for **T1** and 3.14 for **T2** on MIPs fabricated from methacrylic acid. Preliminary microbiological evaluations using *Chromobacterium violaceum* ATCC 12472 reveal that the MIPs prepared from 2-hydroxyethyl methacrylate effectively inhibited violacein production by up to 78.2% at 5.0 mg·mL^−1^, consistent with quorum sensing interference. These findings highlight the feasibility of employing molecular imprinting to target autoinducer-2 analogs, introducing a novel synthetic strategy for disrupting bacterial communication. This further suggests that molecular imprinting can be leveraged to develop potent quorum-sensing inhibitors, an approach that offers translational potential as an alternative to conventional antimicrobial strategies to mitigate biofilm-associated infections.

## 1. Introduction

Molecularly imprinted polymers (MIPs) are a well-known class of materials widely utilized in the separation sciences due to high selectivity, resulting from the formation of binding cavities that are complementary in size, shape and chemical functionality to the template used during synthesis [1,2,3]. MIPs are used as sorbents for specific molecules or selectors for enantioselective resolution [4,5]. They are also applied in molecular sensing, serving as the recognition component of the sensor [6]. Recent advancements in the field of MIPs are largely attributed to the adoption of surface imprinting techniques and the development of imprinting protocols that allow the process to proceed in an aqueous environment, opening new opportunities to apply MIPs [7,8].

One emerging biomedical application is the sequestration of quorum sensing (QS) molecules to attenuate bacterial cell communication. This is achieved by reducing the local concentration of signaling molecules below the threshold required for QS-dependent gene expression [9]. The regulation, modification or inhibition of QS can aid disease control via various QS molecules recognized as autoinducers.

Among the known autoinducers, derivatives of N-acyl homoserine lactones (AHL), whose acyl chain length and headgroup chemistry dictate species-specific signaling, are produced by more than 50 Gram-negative bacterial species. For example, *Aeromonas hydrophilia* synthesizes N-butyryl-L-homoserine lactone and N-hexanoyl-L-homoserine lactone, whereas *Pseudomonas aeruginosa* produces N-butyryl-L-homoserine lactone and N-3-oxododecanoyl-L-homoserine lactone. In Gram-positive bacteria, QS commonly involves oligopeptide signaling molecules operating via a two-component system comprising a membrane-bound sensor kinase and cytoplasmic transcription factors [10]. N-acyl homoserine lactones and oligopeptides are collectively referred to as type 1 autoinducers [11,12,13,14].

**Scheme 1:**
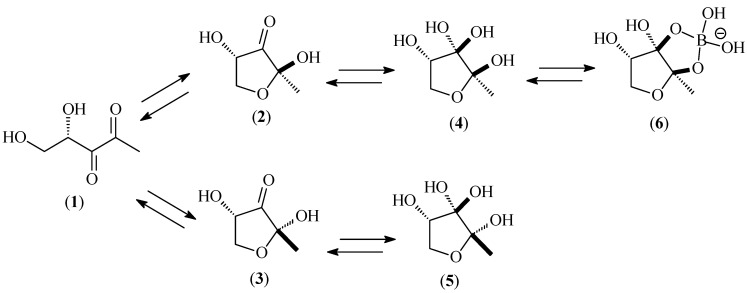
The parent compound (**1**) and various forms of autoinducer-2 (**2**–**6**) in an aqueous environment.

Another widely conserved QS system involves autoinducer-2 (AI-2). AI-2 is a unique, boron-dependent signaling molecule derived from the Lux-mediated cleavage of S-ribosyl-homocysteine, resulting in (S)-4,5-dihydroxy-2,3-pentanedione (DPD), which spontaneously cyclizes into a dynamic equilibrium of furanone derivatives (Scheme 1). In aqueous environments, DPD derivatives further interconvert into hydrated cyclic forms, such as tetrahydroxytetrahydrofurans, which under borate complexation yield (2S,4S)-2-methyl-2,3,3,4-tetrahydroxytetrahydrofuranborate, the primary AI-2 signal molecule in *Vibrio harveyi* and (2S,4S)-2-methyl-2,3,3,4-tetrahydroxytetrahydrofuran found in *Salmonella typhimurium* [15]. AI-2 serves as a universal signaling molecule mediating interspecies and intraspecies communication [16], distinguishing it from species-specific signals such as AHLs, which predominantly regulate intraspecies communication [17]. This AI-2 system is found in over 60 bacterial species, and it interacts with at least four receptor types: LuxP, LsrB, CahR-type receptors containing the dCache_1 domain and YeaJ-type receptors containing the GAPES1 domain [12,18]. In Gram-negative *Vibrio harveyi*, AI-2 binds to the periplasmic protein LuxP, forming a LuxP-AI-2 complex that allosterically activates the membrane-bound histidine kinase receptor LuxQ. Upon AI-2 binding, LuxO undergoes dephosphorylation, switching from a kinase to a phosphatase mode. This alteration decreases phosphorylation of the response Lux Ocia intermediary proteins, LuxU and LuxO, depressing RNA transcription that culminates in the activation of LuxR and the induction of bioluminescence and other QS-regulated genes [18]. In contrast, in *Salmonella typhimurium* and *Escherichia coli*, AI-2 is recognized by LsrB, a high-affinity periplasmic binding protein of the luxS-regulated (Lsr) ABC transporter system [19]. Following AI-2 binding, LsrB facilitates its internalization into the cytoplasm via the LsrACDB transporter [20]. Once internalized, AI-2 is phosphorylated by LsrK, yielding phosphor-AI-2, which binds to the LsrR repressor, triggering dissociation from DNA and activation of the Lsr operon, thus enhancing AI-2 uptake and QS gene expression [21,22,23]. This feedback loop allows AI-2 levels to serve as a proxy for cell density and interspecies presence. The LsrB receptor has been found in Enterobacteriaceae, Rhizobiaceae, Bacillaceae and Clostridia species [24,25]. The CahR-type and YeaJ-type have been recently identified as AI-2 receptors [13,14]. For example, in *Pseudomonas aeruginosa* PAO1, AI-2 binds to the dCache_1 domain of the chemoreceptors PctA and TlpQ, causing both chemotaxis and biofilm formation [13]. The interaction of AI-2 and the YeaI-type receptor is seen in *Salmonella enterica* serovar Typhimurium, where the binding of the QS molecule and bile salts to the GAPES1 portion causes an increase in c-di-GMP synthesis, reducing the activity of the T3SS1-genes and the invasion of epithelial cells [26,27,28]. Importantly, the capacity of AI-2 to engage multiple structurally diverse receptors, including LuxP (boron-dependent furanone sensing), LsrB (phosphorylation-mediated derepression) and the recently described dCache_1 and GAPES1 domain-containing receptors, confers this system with broad host range and adaptive flexibility. Such universality makes AI-2 a compelling target for polymer-based quorum quenching, particularly in polymicrobial environments or bacterial communities with cryptic QS hierarchies.

It must be emphasized that in recent years, alternative pathways of QS have been explored, identifying other molecules that are important to bacterial communication ecosystems [29,30]. QS-regulated biofilm formation plays a key role in the persistence and antimicrobial resistance of multiple microbial infections, contributing significantly to chronic and recurrent clinical conditions.

To combat bacterial infections, various probiotics, enzymes, phytochemical compounds, synthetic inhibitors and polymeric materials were explored to prevent biofilm formation [31]. Among them, MIPs were investigated as a novel class of materials that inhibit attachment of the bacterial cell to the surface, disrupt the biofilm formation, capture the autoinducer molecules (mostly derivatives of AHLs) [32,33,34] and hydrolyze the signaling compounds [35]. For example, Ma and co-workers [32] designed an MIP to specifically capture a QS autoinducer, viz., N-(3-oxododecanoyl)-L-homoserine lactone, to inhibit the biofilm formation of *Pseudomonas aeruginosa*. Various synthetic parameters were optimized to develop an MIP with specificity, comprising itaconic acid and ethylene glycol dimethacrylate, with a high adsorption affinity for N-3-oxododecanoyl-L-homoserine lactone and an imprinting factor (IF) of 1.68. However, it was observed that the MIP synthesized from 2-hydroxyethyl methacrylate and ethylene glycol dimethacrylate was characterized by a fivefold higher adsorption capacity and a 65% reduction in biofilm formation.

In another interesting study, multiple functional monomers were used because various functional groups that are present in different monomer molecules interacted with different components of the template, enhancing the stabilization of the pre-polymerization complex. This is highly beneficial when polypeptides are used as the template, leading to improved imprinting. For example, Motib and co-workers [33] designed an MIP to modulate QS in Gram-positive bacteria, targeting signaling peptide PhrA10. In the designing step, a mixture of different functional monomers with various functionalities was used to prepare a linear MIP. The MIP was designed from four functional monomers used in combination with the pre-polymerization mixture, viz., acrylamide, acrylic acid, N-tert-butylacrylamide and N-(3-aminopropyl)methacrylamide. Acrylic acid and N-(3-aminopropyl)methacrylamide formed ionic interactions with the template, PhrA10. Acrylamide was selected to form hydrogen bonds with the template, and N-tert-butylacrylamide improved interactions of the hydrophobic regions of the template. The effect of the linear MIP on virulence was further confirmed by in vivo evaluation in a mouse model against a lethal microbial infection of pneumococcal pneumonia. The results revealed that the linear MIP prevented the translocation of pneumococci from the lungs to the blood, confirming that the MIP selectively interfered with QS signals.

Another signaling molecule is 2-heptyl-3-hydroxy-4(1H)-quinolone. In a study by Tajani and co-workers [34], structural analogs of the target signaling molecule were used due to the low thermal stability and high cost of the native signal, with analogs selected to preserve key recognition features, such as hydrogen bonding and steric fit. Methacrylic acid, vinylbenzene and ethylene glycol dimethacrylate were used to prepare an MIP in the presence of a structural analog of the target signaling molecule, viz., 3-hydroxy-2-methyl-4(1H)-quinolone. However, because of the low solubility of the template, 1-hexane sulfonic acid sodium salt was used as the phase-transferring agent. The results indicated a significant difference in biofilm inhibition between the MIP (67%) and non-imprinted polymer (NIP, 11%). These examples collectively demonstrate that effective inhibition of QS via molecular imprinting requires strategic design—including appropriate template analogs, complementary functional monomers and optimized pre-polymerization conditions—to ensure high selectivity, stable binding interactions and bioactivity in relevant biological environments. However, it must be emphasized that a survey of the literature reveals a scarcity of MIPs designed to capture other signaling molecules, such as AI-2.

Therefore, in this study, a novel MIP was prepared and analyzed to capture AI-2 molecules. The synthetic pathway to form AI-2 is presented in Scheme 1. It consists of the parent compound (S)-4,5-dihydroxypentanedione (**1**) that exists in an aqueous environment in equilibrium with two diastereomeric cyclic forms (**2** and **3**). These compounds can be hydrated further to 2,3,3,4-tetrahydroxytetrahydrofuranes (**4** and **5**). In the presence of boron, the (2S,4S)-2-methyl-2,3,3,4-tetrahydroxytetrahydrofuranborate (**6**) is formed [35].

The AI-2 molecules interact with receptors LuxP and LsrB, but the specificity is rather high. Hence, only the LuxP receptor binds to (2S,4S)-2-methyl-2,3,3,4-tetrahydroxytetrahydrofuranborate (**6**). The low stability, lack of commercial availability and laborious in vitro synthetic protocol of (2S,4S)-2-methyl-2,3,3,4-tetrahydroxytetrahydrofuranborate (**6**) hinder the use of this compound as a template for molecular imprinting [36]. The structural analogs of (2S,4S)-2-methyl-2,3,3,4-tetrahydroxytetrahydrofuranborate (**6**) were therefore explored in this study as a dual-template strategy, using (3R,4S)-tetrahydro-3,4-furandiol and (R/S) 2,2-dimethyl-1,3-dioxolane-4-methanol.

The dual- or multiple-template approaches to produce MIPs have recently been more frequently investigated as an attractive alternative to single-template systems [37]. The main advantages of dual- or multiple-template approaches can be identified as follows: higher adsorption capacities and improved cross-selectivity in the separation processes, lower limits of detection in the detection methods [38,39,40], more effective targeting of different domains in the cell membranes and the combination of precise targeting with the delivery of drugs in clinical or pharmaceutical applications [41,42]. It is also important to underline that the use of two or more templates during the preparation step significantly affects the morphology of the resulting material, predominantly providing higher extension of the surface of MIP, which, as a consequence, results in higher adsorption capacity [43]. Furtado and co-workers compared the physicochemical parameters of MIPs obtained using single- and dual-template strategies. In that study, MIPs were developed using supercritical carbon dioxide technology, producing an MIP single-templated (st-MIP) by L-leucine or a double-templated MIP (dt-MIP) by a mixture of L-leucine and L-lysine. The resulting materials were used for the selective separation process of amino acids. The analysis of morphology parameters revealed that the dt-MIP was characterized by significantly a higher average diameter of particles (2.72 ± 0.25 µm to 1.72 ± 0.22 µm for st-MIP), a higher specific surface area (58.56 ± 2.86 m^2^·g^−1^ to 41.97 ± 1.34 m^2^·g^−1^ for st-MIP), a higher pore volume (0.27 ± 0.01 cm^3^·g^−1^ to 0.34 ± 0.01 cm^3^·g^−1^ for st-MIP) and lower pore size (25.56 ± 0.80 nm to 22.79 ± 1.13 nm for st-MIP). These parameters affected the adsorption behavior of the resulting MIPs, with adsorption capacities as follows: L-leucine on st-MIP equal to 163 ± 23 mg·g^−1^, L-leucine on dt-MIP equal to 135 ± 3 mg·g^−1^, L-lysine on dt-MIP equal to 193 ± 10 mg·g^−1^ and a mixture of L-leucine and L-lysine on dt-MIP equal to 216 ± 31 mg·g^−1^. The model was extended for the analysis of the adsorption of amino acids in the presence of a multi-component system, confirming the ability of the dt-MIP to selectively recognize its template molecules, even in the presence of a protein [43].

In this study, to predict the effectiveness of MIPs to adsorb the target molecule (2S,4S)-2-methyl-2,3,3,4-tetrahydroxytetrahydrofuranborate (**6**), molecular modeling was used. The resulting MIPs were evaluated in terms of its adsorption capacity towards the template molecules and selectivity. Microscopic and spectroscopic methods were employed for the physicochemical characterization of the MIPs prepared. Finally, the preliminary analysis of the MIPs in a relevant microbiological environment was carried out to assess their ability to modulate quorum sensing. The rationale aligns with evidence from the multi-template imprinting literature, where dual-template systems have been shown to enhance adsorption site diversity and cross-reactivity towards families of structurally related targets, a key feature when targeting interconverting AI-2 furanones. To our knowledge, this is the first report applying such a dual-template molecular mimicry approach to AI-2, potentially enabling broader applicability in interspecies quorum quenching.

## 2. Results and Discussion

### 2.1. Influence of Functional Monomer Components on MIP Preparation

**Figure 1:**
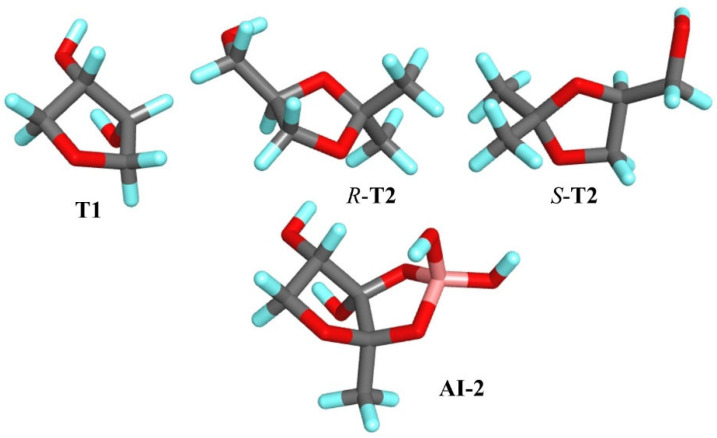
Molecular structures (3D models) of **T1**, **T2** and AI-2 molecules. Oxygen atoms are shown in red, hydrogen atoms in light blue, carbon atoms in grey, and boron atom in salmon pink.

The composition of MIPs plays a crucial role in the adsorption parameters governing the specificity of MIPs. In this study, seven MIPs of different composition were synthesized using various functional monomers, differing in terms of physicochemical properties, viz., methacrylic acid (**1**), itaconic acid (**2**), 4-vinylbenzoic acid (**3**), 2-hydroxyethyl methacrylate (**4**), glycidyl methacrylate (**5**), N-isopropylacrylamide (**6**) and 1-allyl-2-thiourea (**7**). Considering the instability of AI-2 and the possibility that in an in vivo environment, an equilibrium of various compounds is present (as presented in Scheme 1), two different templates were used to fabricate MIPs, viz., (3R,4S)-tetrahydro-3,4-furandiol (**T1**) or (R/S) 2,2-dimethyl-1,3-dioxolane-4-methanol (**T2**), acting as the structural analogs of AI-2. To support the structural similarity between the **T1**/**T2** molecules and the AI-2 molecule, a comparative analysis of key structural and physicochemical parameters was conducted. First, it was observed that both **T1** and AI-2 contain two –OH groups positioned in comparable spatial orientations (Figure 1), which may contribute to similar hydrogen bonding patterns and interaction modes with the target adsorption sites in the resulting MIP.

Additionally, van der Walls’ molecular volumes and surface areas were calculated. The results reveal that the van der Walls molecular volume of AI-2 is equal to 342.79 Å^3^, while that of the *R*-**T2** isomer is 328.76 Å^3^, indicating a high degree of similarity in their molecular size. Similarly, the van der Walls’ surface area of AI-2, equal to 176.30 Å^2^, is comparable to that of the *S*-**T2** isomer (160.75 Å^2^), further supporting the spatial similarity. Moreover, the charge distribution in the fragment of AI-2 structurally resembling **T1** exhibits a similar electrostatic potential profile. The region displays comparable partial charge localization, with electron-rich (negative) and electron-deficient (positive) zones corresponding spatially across both molecules (Figure S1 in the Supplementary Materials). These quantitative parameters—molecular volume, surface area, functional group distribution and charge topology—collectively could reinforce the classification of **T1** and **T2** as structural analogs of the AI-2 molecule.

MIPs were prepared using each template molecule, as well as their equimolar mixture. The obtained MIPs (and NIPs) were evaluated, and the adsorption of **T1** or **T2** on the resulting MIPs and NIPs was analyzed. The adsorption capacity, *B*, of **_T1_**MIP**1**–MIP**7**, **_T2_**MIP**1**–MIP**7**, **_T1_**_/_**_T2_**MIP**1**–MIP**7** and NIP**1**–NIP**7** were calculated according to Equation (1), and IFs were calculated according to Equation (2). The results are presented in Table S2 in Supplementary Materials.

The study was specifically designed for polymers constructed from acidic monomers because the protonation changes are expected to play a significant role in the analyte adsorption process. The protonation state of the monomer residues is crucial because it affects the nature and strength of interactions formed between the analyte and the polymer cavity [44,45]. In this study, the p*K*a values of **T1** and **T2** are 13.6 and 14.2, respectively. Under both pH conditions studied, these compounds remain in the neutral form. Therefore, changes in the analyte/template ionization state do not affect the observed adsorption properties. The observed differences could be attributed solely to the pH-dependent ionization state of the monomer residues within the polymer matrix.

The results reveal that the adsorption of **T1** on all tested MIPs at pH 3.5 varied in the range between 15.0 ± 2.5 µg∙g^−1^ and 40.7 ± 6.9 µg∙g^−1^ for **_T1_**_/**T2**_MIP**7** and **_T1_**MIP**7**, respectively, and the adsorption of **T1** on all tested MIPs in pH 8.5 varied between 8.6 ± 0.5 µg∙g^−1^ and 39.7 ± 2.5 µg∙g^−1^ for **_T1_**MIP**6** and **_T1_**MIP**1**, respectively. Comparing the results of the adsorption of **T1** on MIPs to the adsorption of **T1** on NIPs, the highest specificity was observed for **_T1_**MIP**1** at pH 8.5, with an imprinting factor (IF) = 3.36. This means that the highest selectivity towards **T1** was noted for the MIP prepared in the presence of **T1** as the template. However, it can be found that all MIPs fabricated from acidic monomers, viz., methacrylic acid, itaconic acid or 4-vinylbenzoic acid, revealed selectivity towards **T1** in the range of IFs between 1.26 and 1.97 at pH 3.5, as well as between 1.44 and 3.36 at pH 8.5. It can be found that the MIP fabricated from 2-hydroxyethyl methacrylate was characterized by its specificity at pH 3.5.

The adsorption of **T2** on all tested MIPs at pH 3.5 varied in the range between 2.46 ± 0.5 µg∙g^−1^ and 6.26 ± 0.80 µg∙g^−1^ for **_T1_**_/**T2**_MIP**6** and **_T2_**MIP**1**, respectively, and the adsorption of **T2** on all tested MIPs in pH 8.5 varied between 3.76 ± 0.29 µg∙g^−1^ and 7.48 ± 0.57 µg∙g^−1^ for **_T2_**MIP**7** and **_T1_**_/**T2**_MIP**6**, respectively. Comparing the results of the adsorption of **T2** on MIPs to the adsorption of **T2** on NIPs, the highest selectivity was observed for **_T2_**MIP**1** at pH 3.5 with an IF = 3.14. These findings mean that the highest selectivity towards **T2** was noted for the MIP prepared in the presence of **T2** as the template. However, it can be found that only MIPs fabricated from acidic monomers, viz., methacrylic acid and itaconic acid, reveal selectivity towards **T2**, and this phenomenon was more emphasized at pH 3.5 than at pH 8.5.

To sum up, it could be concluded that **T1** is more specifically adsorbed on the MIPs fabricated in the presence of **T1** and that **T2** is more specifically adsorbed on the MIPs fabricated in the presence of **T2**. The physicochemical nature of the polymer composition plays an important role in the specificity.

### 2.2. In Silico Validation of the Imprinting Process

To elucidate the molecular recognition mechanisms and adsorption properties of the synthesized MIPs, computational simulation studies were performed. Simplified models of pre-polymerization complexes (PCs), composed of monomers and template molecules, were constructed to ensure the simulations remained both cost- and time-efficient. A detailed analysis of the interactions within the pre-polymerization mixtures between the template molecules (**T1**, *R*-**T2** or *S*-**T2**) and the monomers was carried out. Additionally, the calculation of complexation energy (*ΔE_C_*) values, as presented in Table 1, was conducted according to Equation (3). Since the **T2** was used in racemic form in the experimental studies, both enantiomers (*R*-**T2** and *S*-**T2**) were included in the computational models to evaluate their individual interactions and assess the influence of the enantiomeric form on the adsorption behavior of the MIPs.

**Figure 2:**
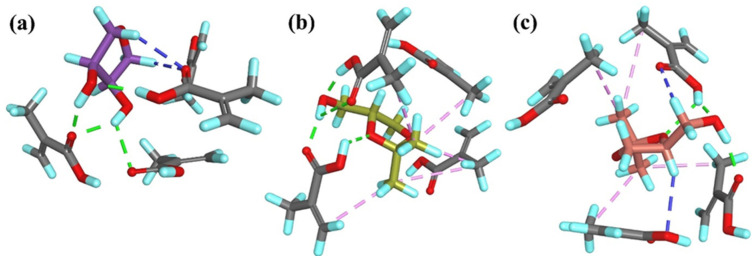
Optimized models of pre-polymerization complexes: **_T1_**PC**1** (**a**), *_R_*_-**T2**_PC**1** (**b**) and *_S_*_-**T2**_PC**1** (**c**). Oxygen atoms are shown in red, hydrogen atoms in light blue, carbon atoms in monomers in grey and carbon atoms in template molecules in purple (**a**), yellow (**b**) or salmon pink (**c**). The classical hydrogen bonds are indicated by green dashed lines, the non-classical hydrogen bonds by blue dashed lines and hydrophobic interactions by pink dashed lines.

In the **_T1_**PC**1** system, four classical hydrogen bonds were formed between both –OH groups of **T1** and three monomer molecules. Additionally, two non-classical hydrogen bonds were observed between the H atoms of the –CH_2_– groups from the template and the O atom from one monomer molecule (Figure 2a). In the *_R_*_-**T2**_PC**1** and *_S_*_-**T2**_PC**1** systems, four and three classical hydrogen bonds, respectively, were identified, involving either the –OH group or the ether O atom from the template and two monomer molecules. Both systems also exhibited hydrophobic interactions between the –CH_3_ groups of the template and the alkyl or vinyl groups of the monomers (Figure 2b,c). Moreover, in *_S_*_-**T2**_PC**1**, similarly to **_T1_**PC**1**, two non-classical hydrogen bonds were identified between the H atoms of the –CH_2_– or –CH– groups from the template and the O atoms from the monomers. The overall similarity in the number and type of interactions within all PC**1** complexes was consistent with the small differences observed in their *ΔE_C_* values (Table 1).

**Figure 3:**
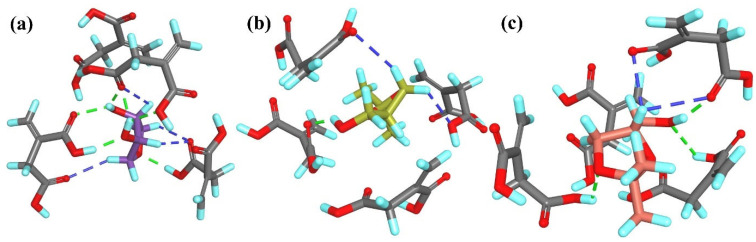
Optimized models of pre-polymerization complexes: **_T1_**PC**2** (**a**), *_R_*_-**T2**_PC**2** (**b**) and *_S_*_-**T2**_PC**2** (**c**). Oxygen atoms are shown in red, hydrogen atoms in light blue, carbon atoms in monomers in grey and carbon atoms in template molecules in purple (**a**), yellow (**b**) or salmon pink (**c**). The classical hydrogen bonds are indicated by green dashed lines and the non-classical hydrogen bonds by blue dashed lines.

In the **_T1_**PC**2** system, six classical hydrogen bonds were formed between the –OH groups or the ether O atom from the template and the –COOH groups from the monomer molecules. Additionally, four non-classical hydrogen bonds were observed between the –CH_2_– or –CH– groups from the template and the monomers (Figure 3a). In the *_R_*_-**T2**_PC**2** and _S-**T2**_PC**2** systems, one and three classical hydrogen bonds, respectively, were identified, involving the –OH group or the ether O atom from the template and one or three monomer molecules. Like **_T1_**PC**2**, two non-classical hydrogen bonds were observed in both *_R_*_-**T2**_PC**2** and *_S_*_-**T2**_PC**2**: in the former, between the –CH_2_– group from the template and two monomer molecules; in the latter, between the –CH– group of the ring from the template and one monomer molecule (Figure 3b,c). Due to the presence of two carboxyl groups in the itaconic acid monomer, only hydrogen bonds were formed in the PC**2** systems. The high polarity and hydrophilicity of the carboxyl groups rendered hydrophobic interactions negligible in these systems. Consequently, the *ΔE_C_* values obtained in the computational studies were lower than those for other systems (Table 1), indicating that PC**2** complexes are stabilized by strong hydrophilic interactions between the monomers.

**Figure 4:**
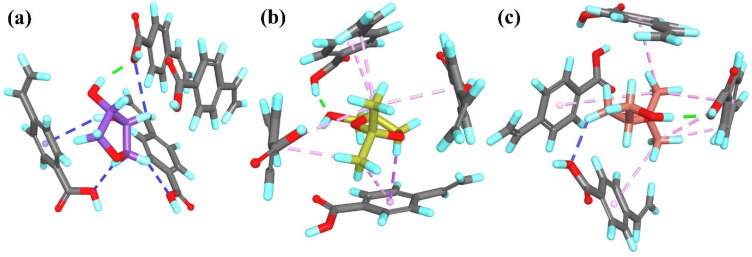
Optimized models of pre-polymerization complexes: **_T1_**PC**3** (**a**), *_R_*_-**T2**_PC**3** (**b**) and *_S_*_-**T2**_PC**3** (**c**). Oxygen atoms are shown in red, hydrogen atoms in light blue, carbon atoms in monomers in grey and carbon atoms in template molecules in purple (**a**), yellow (**b**) or salmon pink (**c**). The classical hydrogen bonds are indicated by green dashed lines, the non-classical hydrogen bonds by blue dashed lines and hydrophobic interactions by pink or purple dashed lines.

Due to the hydrophobic nature of the aromatic rings in the monomers used in PC**3** systems, the types and contributions of interactions differ between **T1** and **T2** templates. In particular, the presence of alkyl groups in **T2** molecules allows for the formation of additional hydrophobic interactions with the monomers. Moreover, the shape and spatial arrangement of monomers in the **_T1_**PC**3** system differ from those in the **_T2_**PC**3** systems, likely because of the hydrophobic character of the monomers. In the **_T1_**PC**3** complex, one classical and four non-classical hydrogen bonds formed between the –OH or the –CH_2_– groups from the template and the carboxyl groups or aromatic moieties from three monomer molecules (Figure 4a). In contrast, the *_R_*_-**T2**_PC**3** and *_S_*_-**T2**_PC**3** systems exhibited numerous hydrophobic interactions. In *_R_*_-**T2**_PC**3**, five π-alkyl interactions were observed between the –CH_3_ groups from the template and the aromatic rings from all monomer molecules, along with one alkyl–alkyl interaction between the –CH_3_ group from the template and the vinyl group from the monomer and one π-sigma interaction between the –CH_2_– group from *R*-**T2** and the aromatic ring from the monomer. Similarly, in *_S_*_-**T2**_PC**3**, five π-alkyl interactions and one alkyl–alkyl interaction were formed between the –CH_3_ groups from the template and the aromatic rings or vinyl groups from all monomer molecules. Additionally, both **_T2_**PC**3** systems featured one classical hydrogen bond involving the –OH group from the template, while *_S_*_-**T2**_PC**3** also exhibited one non-classical hydrogen bond between the –CH_2_– group from the template and the monomer (Figure 4b,c). The spatial arrangement of monomers and the nature of intermolecular interactions are likely responsible for the lower *ΔE*_C_ value observed for the **_T1_**PC**3** system compared to the analogous systems with **T2** templates (Table 1).

**Figure 5:**
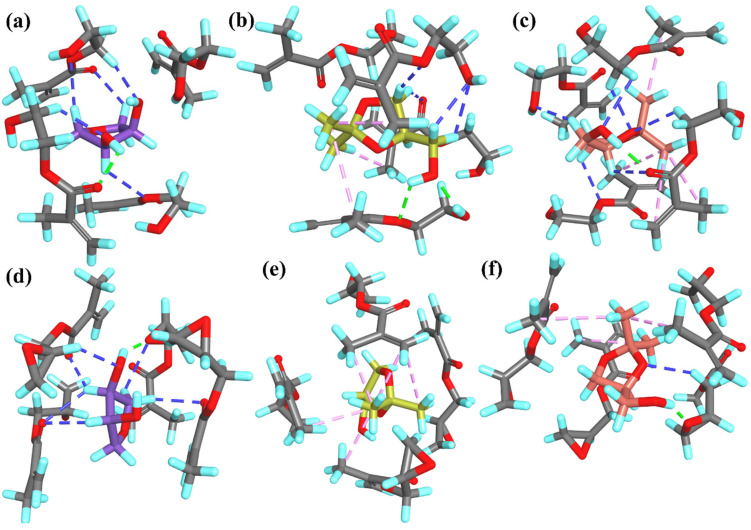
Optimized models of pre-polymerization complexes: **_T1_**PC**4** (**a**), ***_R_***_-**T2**_PC**4** (**b**), *_S_*_-**T2**_PC**4** (**c**), **_T1_**PC**5** (**d**), *_R_*_-**T2**_PC**5** (**e**) and *_S_*_-**T2**_PC**5** (**f**). Oxygen atoms are shown in red, hydrogen atoms in light blue, carbon atoms in monomers in grey and carbon atoms in template molecules in purple (**a**,**d**), yellow (**b**,**e**) or salmon pink (**c**,**f**). The classical hydrogen bonds are indicated by green dashed lines, the non-classical hydrogen bonds by blue dashed lines and hydrophobic interactions by pink dashed lines.

In the PC**4** and PC**5** systems, which involve ester or ether monomers, the mode of interaction between the template molecules and the monomers was generally similar and resembled those observed in the PC**1** and PC**3** models, where hydrophobic interactions were present in systems containing **T2**. We observed no classical hydrogen bonds in *_R_*_-**T2**_PC**5**, one in **_T1_**PC**4**, *_S_*_-**T2**_PC**4**, **_T1_**PC**5** and *_S_*_-**T2**_PC**5** and two in *_R_*_-**T2**_PC**4**. These interactions involved the –OH groups from the template and either the hydroxyl or ether O atoms from 2-hydroxyethyl methacrylate or the ether O atom from glycidyl methacrylate. Non-classical hydrogen bonds were present in all PC**4** and PC**5** systems, except *_R_*_-**T2**_PC**5**. For systems with **T1**, a greater number of non-classical hydrogen bonds was observed in PC**5** (six) compared to PC**4** (five). In contrast, for models with **T2**, the trend was reversed: four and six non-classical hydrogen bonds were observed in *_R_*_-**T2**_PC**4** and *_S_*_-**T2**_PC**4**, respectively, but none and one in *_R_*_-**T2**_PC**5** and *_S_*_-**T2**_PC**5**, respectively. Non-classical hydrogen bonds were primarily established between the –CH_2_– or –CH– groups from the templates and various O atoms from the monomers in **_T1_**PC**5** and all PC**4** systems. Additionally, in **_T1_**PC**4**, *_S_*_-**T2**_PC**4** and *_S_*_-**T2**_PC**5**, this type of interaction involved the O atoms of the –OH group or the ring from the templates and the –CH_2_– groups from the monomers (Figure 5). Hydrophobic alkyl–alkyl interactions were observed between the template and the monomers in the **_T2_**PC**4** and **_T2_**PC**5** systems, ranging from three interactions (in *_R_*_-**T2**_PC**4** and *_S_*_-**T2**_PC**5**) to five (in *_R_*_-**T2**_PC**5**). In *_R_*_-**T2**_PC**5**, only hydrophobic template–monomer interactions were present; no hydrogen bonds were detected (Figure 5e). In the PC**5** models, the presence of hydrogen bonds contributed to system stabilization, as reflected in the *ΔE_C_* values. **_T1_**PC**5**, which had the highest number of hydrogen bonds, exhibited the lowest *ΔE_C_*, whereas *_R_*_-**T2**_PC**5**, with no hydrogen bonds, showed the highest *ΔE_C_*. Moreover, the presence of the hydrophobic interactions in **_T2_**PC**4** systems may account for the slightly lower *ΔE*_C_ values compared to **_T1_**PC**4** (Table 1). Due to the hydrophilic nature of 2-hydroxyethyl methacrylate, a greater number of hydrogen bonds were observed in **_T2_**PC**4** systems compared to **_T2_**PC**5** systems, which involved less hydrophilic glycidyl methacrylate (Figure 5b,c,e,f).

**Figure 6:**
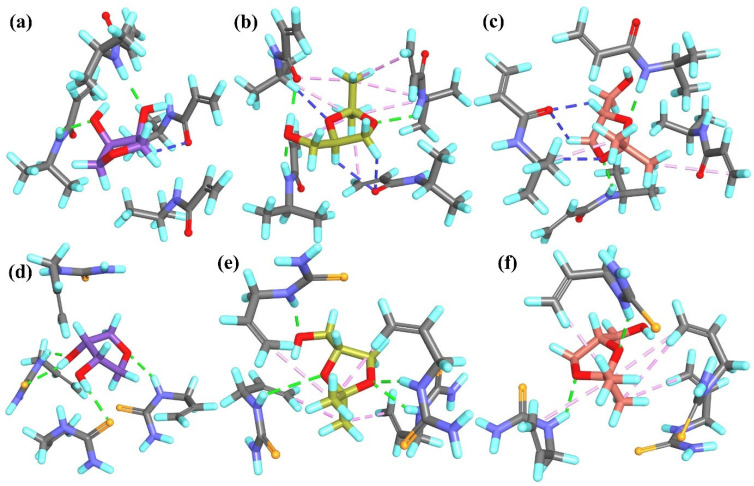
Optimized models of pre-polymerization complexes: **_T1_**PC**6** (**a**), *_R_*_-**T2**_PC**6** (**b**), *_S_*_-**T2**_PC**6** (**c**), **_T1_**PC**7** (**d**), *_R_*_-**T2**_PC**7** (**e**) and *_S_*_-**T2**_PC**7** (**f**). Oxygen atoms are shown in red, hydrogen atoms in light blue, nitrogen atoms in blue, sulfur atoms in orange, carbon atoms in monomers in grey and carbon atoms in template molecules in purple (**a**,**d**), yellow (**b**,**e**) or salmon pink (**c**,**f**). The classical hydrogen bonds are indicated by green dashed lines, the non-classical hydrogen bonds by blue dashed lines and hydrophobic interactions by pink dashed lines.

In the PC**6** and PC**7** models, which involved non-acidic and non-ester monomers, we observed two classical and one non-classical hydrogen bond in the **_T1_**PC**6** system and four classical hydrogen bonds in the **_T1_**PC**7** system. As in the PC**4** systems, the classical hydrogen bonds involved the –OH groups from the template and either heteroatoms or the H atoms attached to heteroatoms in the monomers. Notably, in the **_T1_**PC**7** model, the ether O atom from the template also participated in hydrogen bonding; this system was stabilized exclusively by classical hydrogen bonds. In the **_T1_**PC**6** model, the non-classical hydrogen bond was formed between the –CH– group from the template and the O atom from the monomer (Figure 6a,d). In systems containing **T2** templates, as in PC**4**, hydrophobic interactions between the –CH_3_ groups from the template and monomers were present, in addition to hydrogen bonding. In the PC**6** systems, three or two classical hydrogen bonds were observed in *_R_*_-**T2**_PC**6** and *_S_*_-**T2**_PC**6**, respectively. Both systems exhibited three non-classical hydrogen bonds and seven (*_R_*_-**T2**_PC**6**) or two (*_S_*_-**T2**_PC**6**) hydrophobic alkyl–alkyl interactions. In *_R_*_-**T2**_PC**6**, the –OH group from the template created two hydrogen bonds and the ether O atom one, while in *_S_*_-**T2**_PC**6**, only the ether O atoms from the template participated in hydrogen bonding. The non-classical hydrogen bonds in these systems formed similarly to PC**4** systems, involving the –CH_2_–, –CH– groups or the ether O atom from the template and either the O atom or the –CH– group from the monomers. In the **_T2_**PC**7** models, four or two classical hydrogen bonds were observed for *_R_*_-**T2**_PC**7** and *_S_*_-**T2**_PC**7**, respectively. These involved the –OH group (only in *_R_*_-**T2**_PC**7**) or the ether O atoms from the template and the H atoms of the –NH– groups from the monomers. Additionally, four (*_R_*_-**T2**_PC**7**) and five (*_S_*_-**T2**_PC**7**) hydrophobic alkyl–alkyl type interactions were observed (Figure 6b,c,e,f). No non-classical hydrogen bonds were formed in the PC**7** systems.

Following the simulation of pre-polymerization complex formation, lower *ΔE_C_* values were observed for systems composed of acidic monomers across all studied templates. This suggests that acidic monomers can form more stable pre-polymerization mixtures, potentially leading to better-defined polymeric cavities. This observation was corroborated by the experimental results: adsorption analyses show higher adsorption capacity (*B*) and IF values for MIPs synthesized from acidic monomers when interacting with the studied compounds. Additionally, lower *ΔE_C_* values were recorded for **_T1_**PC**1** to **_T1_**PC**3** systems compared to the corresponding *R*-**T2** and *S*-**T2** systems. This trend was not observed in other PC systems constructed with non-acidic monomers. The lower *ΔE_C_* values for acidic systems with **T1** are likely due to the presence of two hydroxyl groups in **T1**, compared to only one in **T2**. These –OH groups enable additional interactions with acidic monomers. Additionally, differences in charge distribution between the **T1** and **T2** template molecules may also play a role in their interactions with monomers (Figure S1 in Supplementary Materials). Interestingly, in systems with acidic monomers, slightly lower *ΔE_C_* values were observed for models with the *R*-enantiomer of **T2** compared to those with the *S*-enantiomer.

### 2.3. Physicochemical Characterization of MIPs

#### 2.3.1. Morphological Investigations

**Figure 7:**
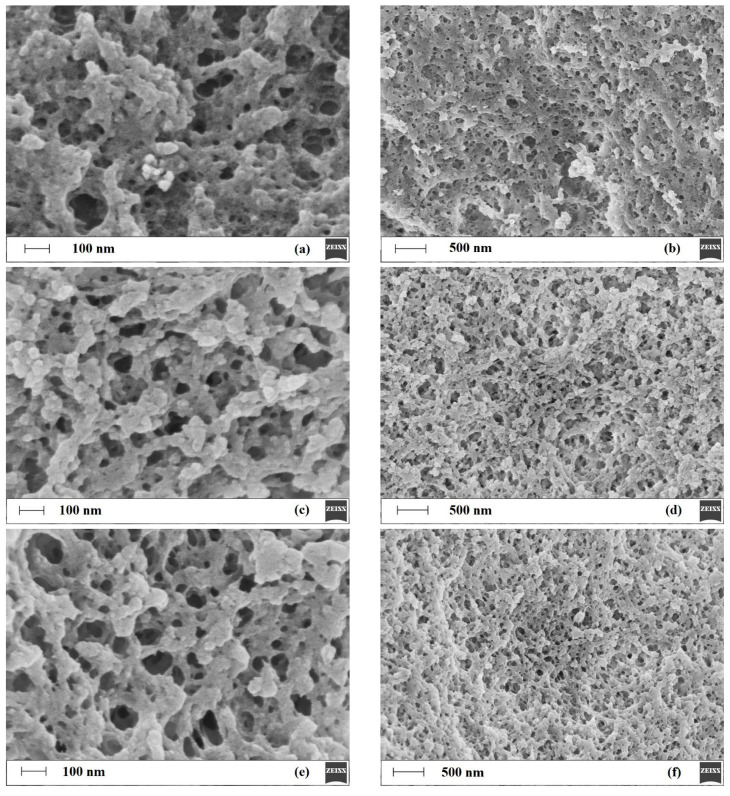

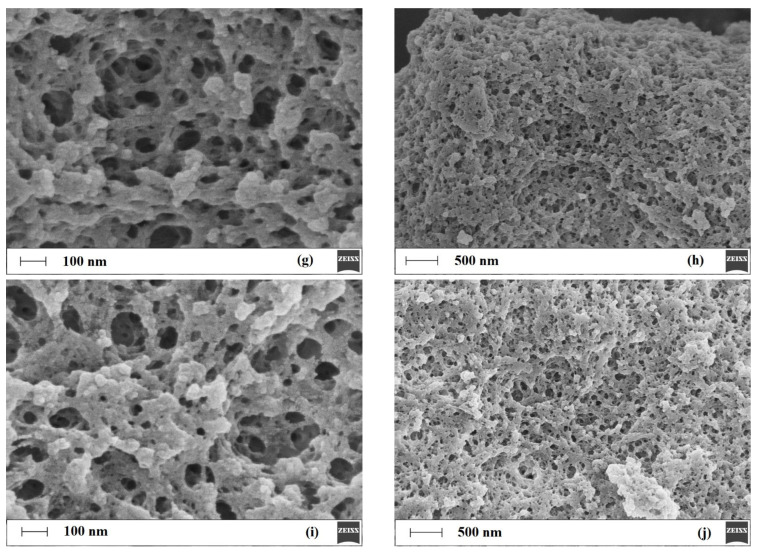
Micrographs of **_T1_**MIP**1** (**a**,**b**), **_T1_**_/**T2**_MIP**1** (**c**,**d**), NIP**1** (**e**,**f**), **_T1_**MIP**7** (**g**,**h**) and NIP**7** (**i**,**j**).

The particle morphology, described as a macro- and microporous structure and specific surface area, is an important parameter related to the specificity of MIPs. Field emission scanning electron microscopy (FE-SEM) was employed to observe the surface of the particles of **_T1_**MIP**1** (the polymer characterized by highest selectivity towards **T1**), **_T1_**_/**T2**_MIP**1** (the polymer characterized by highest selectivity towards **T1** and **T2**), **_T1_**MIP**7** (the polymer characterized by low selectivity towards **T1** and **T2**) and references NIP**1** and NIP**7**. Figure 7 presents micrographs of the analyzed MIPs and NIPs.

As observed in Figure 7, the particles are typical for polymers prepared by bulk polymerization [46]. The micrographs of MIPs and NIPs reveal irregular entities with a diameter of about 20–30 μm. Further magnification reveals only subtle differences in the surface morphologies of the tested materials. It could be found that **_T1_**MIP**1** (Figure 7a,b), **_T1_**_/**T2**_MIP**1** (Figure 7c,d) and **_T1_**MIP**7** (Figure 7g,h) are characterized by a slightly more extended surface and a higher number of macropores when compared to NIP**1** (Figure 7e,f) and NIP**7** (Figure 7i,j).

To discuss in more detail the impact of the use of a dual-template approach during the imprinting process on the morphology of the resulting materials, the sorption of nitrogen at 77 K was measured to determine the specific surface area of **_T1_**MIP**1**, **_T1_**_/**T2**_MIP**1**, NIP**1**, **_T1_**MIP**7** and NIP**7**. Data was fitted to the BET (Brunauer–Emmett–Teller) and BJH (Barrett–Joyner–Halenda) models. Firstly, the nitrogen adsorption isotherm (BET isotherm) was analyzed [47].

**Figure 8:**
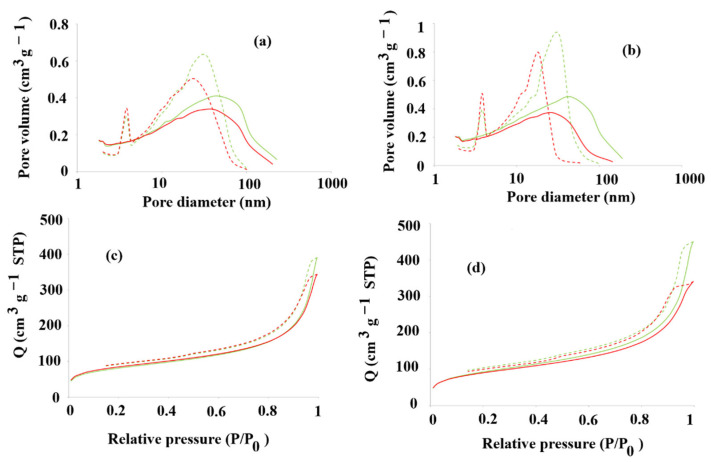
Pore size distributions calculated using the BJH model on both the adsorption and the desorption branches of the isotherm of (**a**) **_T1_**_/**T2**_MIP**1** and NIP**1** and (**b**) **_T1_**MIP**7** and NIP**7**, together with the nitrogen adsorption isotherms for (**c**) **_T1_**_/**T2**_MIP**1** and NIP**1** and (**d**) **_T1_**MIP**7** and NIP**7**; adsorption lines are continuous; desorption lines are dotted; MIP—green, NIP—red (P_0_ is the nitrogen saturation pressure).

Figure 8a–d presents the nitrogen sorption/desorption isotherms of **_T1_**_/**T2**_MIP**1** and NIP**1**, **_T1_**MIP**7** and NIP**7**. The physisorption isotherms of all tested materials reveal intermediate H3 and H4 loops, indicating a narrow slit-like pore system [48]. Then, the total specific surface area (BET isotherm) was determined, together with the cumulative surface area of pores (BJH model) and the volume and the area of micropores (the Harkins–Jura equation) [49,50]. The total specific surface areas of **_T1_**_/**T2**_MIP**1**, NIP**1**, **_T1_**MIP**7** and NIP**7** were equal to 287.1 m^2^∙g^−1^ and 296.4 m^2^∙g^−1^, 330.7 m^2^∙g^−1^ and 318.7 m^2^∙g^−1^, respectively.

#### 2.3.2. Structural Confirmation

To confirm the structure of obtained polymers, infra-red (FT-IR) analysis was employed for the evaluation of **_T1_**MIP**1**, **_T2_**MIP**1**, **_T1_**_/**T2**_MIP**1** and NIP**1** (the polymers characterized by high selectivity). The characteristic vibration peaks, derived from that structural fragments of **_T1_**MIP**1**, **_T2_**MIP**1** and **_T1/T2_**MIP**1** (after the template removal step) could be assigned, respectively, as follows: 3564, 3547 and 3460 cm^−1^ (broad), the –OH stretching vibrations; 2994, 2995, 2995 cm^−1^ and 2960, 2961, 2960 cm^−1^, the stretching vibration of the –CH; 1732, 1735 and 1736 cm^−1^, the –C=O stretching vibration; 1638 cm^−1^ (across all polymers), the stretching of –C=C bonds; 1480 cm^−1^ (across all polymers), the stretching of –CH_2_–CH_2_; 1391, 1392 and 1392 cm^−1^, the stretching of –CH_3_; 1264, 1265, 1265 cm^−1^ and 1163, 1164, 1163 cm^−1^, the C–O–C stretching vibrations. A very similar pattern was observed for NIP**1**: 3555 cm^−1^, 2995 cm^−1^, 2960 cm^−1^, 1732 cm^−1^, 1638 cm^−1^, 1479 cm^−1^, 1392 cm^−1^, 1264 cm^−1^ and 1162 cm^−1^. The difference observed between the spectra of **_T1_**MIP**1**, **_T2_**MIP**1** and **_T1_**_/**T2**_MIP**1** and the spectrum of NIP**1** in the broad range 3300–3600 cm^−1^ could be explained by the formation of interactions between the polymer or an insufficiently removed template. However, it has to be emphasized that the removal of templates was monitored by the LC-MS analysis. The signal intensities for both template molecules significantly decreased below the detection threshold after prolonged extraction. The spectra of **_T1_**MIP**1**, **_T2_**MIP**1**, **_T1_**_/**T2**_MIP**1** and NIP**1** are presented in Figure S2 in the Supplementary Materials.

#### 2.3.3. Selectivity Studies

Considering the application of material, the characterization of designed MIPs in the multicomponent environment is very important. For that purpose, the evaluation of selective adsorption of desired molecules from the mixture of various compounds is necessary to assess the practical potential of materials. In this study, five chemical compounds were selected for the selectivity tests, viz., (R/S) 2,2-dimethyl-1,3-dioxolane-4-methanol (**T2**), (R/S) 2,2-dimethyl-1,3-dioxolane-4-methanamine (**A1**), 2-amine-1-phenylethanol (**A2**), 2-phenylethylamine (**A3**) and 2-(1-cyclohexenyl)ethylamine (**A4**).

For the selectivity studies, the template molecule **T2** was selected, together with its close structural analog **A1**, a compound possessing the same heterocyclic system as **T2** but which possesses the amine group instead of the hydroxy group in the aliphatic chain. The **A2** compound was also selected, as it possesses both hydroxy and amine groups in the aliphatic chain but also contains an aromatic system. The **A3** analog was also chosen, as it comprises an amine group in the aliphatic chain and the aromatic system. Finally, the test compound **A4**, possessing unsaturated bonds in the cyclic ring and the amine group in the aliphatic chain, was also considered.

Taking into account the results of the analysis of adsorption of **T1** and **T2** on **_T1_**MIP**1**–**_T1_**MIP**7**, the polymers **_T2_**MIP**1**–**_T2_**MIP**7**, **_T1_**_/**T2**_MIP**1**–**_T1_**_/**T2**_MIP**7** and all MIPs were selected for the selectivity tests, except for **_T1_**MIP**5**, **_T2_**MIP**5**, **_T1_**_/**T2**_MIP**5** and **_T1_**MIP**7**, **_T2_**MIP**7**, **_T1_**_/**T2**_MIP**7** due to the unfavorable results observed for the adsorption of **T1** and **T2**. The results are presented in Table S3 in the Supplementary Materials. The results reveal that the highest adsorption capacity towards **T2** from the mixture of **T2** and **A1**–**A4** was noted for **_T1_**MIP**2** (10.0 ± 1.3 µg∙g^−1^), but no selectivity towards **T2** in the system containing **A1**–**A4** was observed. Only slightly lower adsorption capacities were noted for **_T2_**MIP**2**, **_T1_**_/**T2**_MIP**2** and **_T1_**MIP**3**, **_T2_**MIP**3** and **_T1_**_/**T2**_MIP**3** with similar very low selectivity patterns. The lowest adsorption capacity towards **T2** was noted for **_T1_**MIP**6** (0.676 ± 0.086 µg∙g^−1^). The results also confirm higher selectivity of **_T2_**MIP**1** towards **T2** but from the mixture of **T2** and **A1**–**A4** when compared to the selectivity of **_T1_**MIP**1** (5.42 ± 0.69 µg∙g^−1^ to 4.35 ± 0.55 µg∙g^−1^, respectively). Surprisingly, the highest selectivity for **T2** from the mixture of **T2** and **A1**–**A4** was observed for **_T1_**MIP**4** and **_T2_**MIP**4**, with the following selectivity factors, which were defined as ratios of the adsorption capacity of **T2** to **A1** or **A2** or **A3** or **A4**, for **_T1_**MIP**4**: **T2**/**A1**, 3.48, **T2**/**A2**, 2.42, **T2**/**A3**, 3.47, **T2**/**A4**, 1.56 and for **_T2_**MIP**4**: **T2**/**A1**, 7.39, **T2**/**A2**, 2.93, **T2**/**A3**, 3.27, **T2**/**A4**, 2.83. The selectivity was significantly lowered for **_T1_**_/**T2**_MIP**4**. Thus, for the preliminary microbiological studies, the following materials were selected: **_T1_**MIP**2**, as the material characterized by the highest adsorption capacity and very low selectivity; **_T1_**MIP**6**, as the material characterized by the lowest adsorption capacity and low selectivity; and **_T1_**MIP**4** and **_T2_**MIP**4** as the materials characterized by high adsorption selectivity towards **T2** in mixtures containing **T2** and its structural analogues **A1**–**A4**. In contrast, the dual-template polymer **_T1/T2_**MIP**4** showed markedly lower selectivity under the same conditions. As a control material, the NIP**2**, characterized by high adsorption capacity, was also included in the microbiological analysis. Moreover, the structure–selectivity relationships among the analytes were investigated employing theoretical analysis. Computational studies were performed for the **_T1_**MIP**1** and **_T1_**MIP**4** systems using the methodology described in our previous works [51]. The MIP**1** system was selected as it was composed of acidic monomers and showed variation in the adsorption capacity towards different analytes. The MIP**4** system was chosen due to its high adsorption selectivity towards **T2** in a mixture, and model cavities of MIPs were constructed based on the **_T1_**PC**1** and **_T1_**PC**4** complex models. The analytes, *R*- and *S*-**T2**, *R*- and *S*-**A1**, *R*- and *S*-**A2**, and **A3** and **A4**, were individually inserted into the model cavity after template molecule removal, and the binding energies (*ΔE_B_*) values, calculated according to Equation (4), are presented in Table 2. The intermolecular interactions were analyzed.

**Figure 9:**
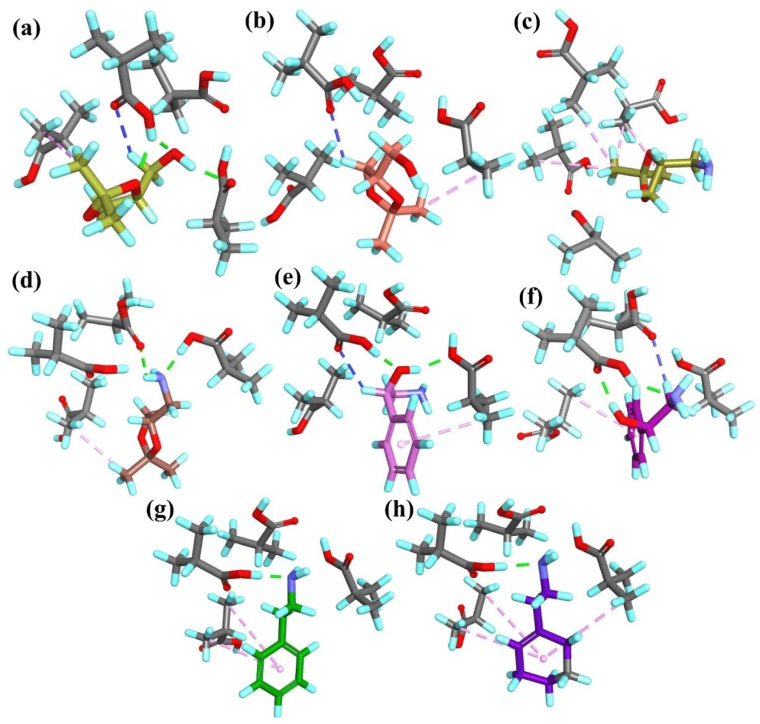
Models of the **_T1_**MIP**1** cavity with analytes bound: *R*-**T2** (**a**), *S*-**T2** (**b**), *R*-**A1** (**c**), S-**A1** (**d**), *R*-**A2** (**e**), *S*-**A2** (**f**), **A3** (**g**) and **A4** (**h**). Oxygen atoms are shown in red, hydrogen atoms in light blue, carbon atoms from monomers in grey and carbon atoms from analyte molecules in yellow (**a**), salmon pink (**b**), mustard (**c**), cocoa (**d**), pink (**e**), purple (**f**), green (**g**) and dark blue (**h**). Classical hydrogen bonds are indicated by green dashed lines, non-classical hydrogen bonds by blue dashed lines and hydrophobic interactions by pink dashed lines.

The *B* of **_T1_**MIP**1** was similar for analytes **T2** and **A1**, and the corresponding *ΔE_B_* values were also comparable and higher than those calculated for **A2**–**A4** (Table 2). *R*-**T2** interacted with the **_T1_**MIP**1** cavity through three classical hydrogen bonds: two formed between the O atom of the –OH group or the ether O atom and the H atom of the –COOH group from the monomer residue and one formed between the H atom of the –OH group and the O atom of the –COOH group. Additionally, one non-classical hydrogen bond and one alkyl–alkyl hydrophobic interaction were observed between *R*-**T2** and the monomer residues (Figure 9a). In contrast, the *S*-**T2** enantiomer formed only one non-classical hydrogen bond and one alkyl–alkyl hydrophobic interaction with the methacrylic acid residues. The spatial arrangement of the two enantiomers within the binding cavity differed significantly (Figure 9b). *R*-**A1** interacted with the **_T1_**MIP**1** cavity solely through four alkyl–alkyl hydrophobic interactions (Figure 9c). On the other hand, *S*-**A1** formed two hydrogen bonds between the N or H atom of the –NH_2_ group and the H or O atom of the –COOH group from the monomer residue. One additional alkyl–alkyl hydrophobic interaction was also observed (Figure 9d). The interaction pattern of *R*-**A2** with **_T1_**MIP**1** was similar to that of *R*-**T2**. *R*-**A2** formed two hydrogen bonds involving the O or H atom of the –OH group and the H or O atom of the –COOH group from the methacrylic acid residues. Additionally, one non-classical hydrogen bond and one π–alkyl hydrophobic interaction were observed (Figure 9e). *S*-**A2** also formed two hydrogen bonds with **_T1_**MIP**1**, but the interacting atoms differed. These involved the N atom of the –NH_2_ group or the H atom of the –OH group from the analyte and the H or O atom of the –COOH group from the methacrylic acid residue. As with *R*-**A2**, one non-classical hydrogen bond and one π–alkyl hydrophobic interaction were also present (Figure 9f). The **A3** and **A4** each formed one classical hydrogen bond between the N atom of the –NH_2_ group and the H atom of the –COOH group from the methacrylic acid residue. Additionally, the **A3** formed two π–alkyl hydrophobic interactions, while the **A4** formed three alkyl–alkyl hydrophobic interactions (Figure 9g and Figure 9h, respectively).

To sum up, the –OH and –NH_2_ groups present in the analyte molecules are primarily responsible for forming interactions during the adsorption process on **_T1_**MIP**1**, mainly through hydrogen bonding with the monomer residues. Only in the cases of *S*-**T2** and *R*-**A1** were these functional groups not involved in the interactions observed during adsorption simulations. All enantiomers of **T2** and **A1**, which contain a similar methyl-substituted ring fragment, formed alkyl–alkyl hydrophobic interactions. However, their spatial arrangement within the cavity differed in each case. This observation suggests that the presence of the ether ring may not be critical for interaction with the acidic polymeric cavity and could contribute to the lower adsorption capacity observed for **_T1_**MIP**1**. Analytes **A2**–**A4**, which contain an aromatic or cyclic ring with unsaturated bonds, interacted with **_T1_**MIP**1** in a comparable manner, despite differences in spatial arrangement (*R*-**A2**). The combination of polar and hydrophilic groups (–OH or/and –NH_2_) with non-polar, hydrophobic aromatic or aliphatic cyclic fragments appears to enhance adsorption capacity in the **_T1_**MIP**1** system. Both enantiomers of the **A2** analyte, which contains both –OH and –NH_2_ groups, interacted with the **_T1_**MIP**1** cavity via –OH, mirroring the interaction pattern of the template molecule **T1**. Only the *S*-enantiomer additionally formed interactions through the –NH_2_ group.

**Figure 10:**
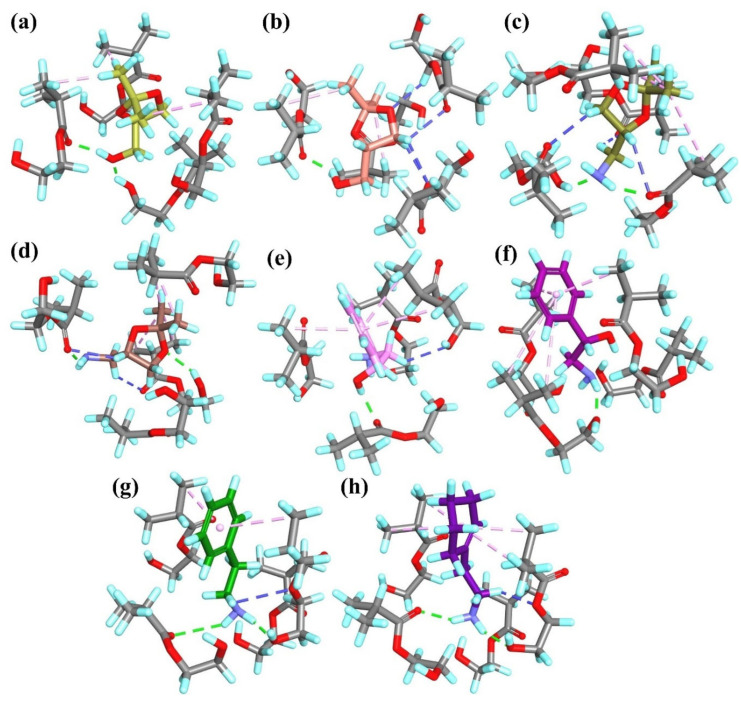
Models of the **_T1_**MIP**4** cavity with analytes bound: *R*-**T2** (**a**), *S*-**T2** (**b**), *R*-**A1** (**c**), *S*-**A1** (**d**), *R*-**A2** (**e**), *S*-**A2** (**f**), **A3** (**g**) and **A4** (**h**). Oxygen atoms are shown in red, hydrogen atoms in light blue, carbon atoms from monomers in grey and carbon atoms from analyte molecules in yellow (**a**), salmon pink (**b**), mustard (**c**), cocoa (**d**), pink (**e**), purple (**f**), green (**g**) and dark blue (**h**). Classical hydrogen bonds are indicated by green dashed lines, non-classical hydrogen bonds by blue dashed lines and hydrophobic interactions by pink dashed lines.

For **_T1_**MIP**4**, the highest adsorption capacities were noted for **T2** and **A4**. However, overall, the adsorption capacities were significantly lower than those for **_T1_**MIP**1**. A similar trend was observed in the *ΔE_B_* values across all analytes: lower *ΔE_B_* values were recorded for **T2** and **A4**, while higher values were obtained for the remaining analytes (Table 2). Additionally, for all chiral analytes, the *S*-enantiomers exhibited higher *ΔE_B_* values than their corresponding *R*-enantiomers. *R*-**T2** interacted with the **_T1_**MIP**4** cavity by forming two hydrogen bonds between the O or H atom of the –OH group and the H or O atom from the hydroxyethyl methacrylate residues, as well as three alkyl–alkyl hydrophobic interactions (Figure 10a). *S*-**T2** formed one hydrogen bond between the H atom of the –OH group and the O atom of the –COO– group from the monomer residue, in addition to four non-classical hydrogen bonds and two alkyl–alkyl hydrophobic interactions (Figure 10b). *R*-**A1** interacted with the **_T1_**MIP**4** cavity through two hydrogen bonds between the N or H atom of the –NH_2_ group and the H or O atoms from the monomer residues. Furthermore, three non-classical hydrogen bonds and four alkyl–alkyl hydrophobic interactions were observed (Figure 10c). *S*-**A1** formed two hydrogen bonds involving the H atom of the –NH_2_ or the ether O atom and the O or H atom from the monomer residues, along with two non-classical hydrogen bonds and four alkyl–alkyl hydrophobic interactions (Figure 10d). *R*-**A2** formed one hydrogen bond between the H atom of the –OH group and the O atom of the –COO– group from the hydroxyethyl methacrylate residue, as well as one non-classical hydrogen bond and three π–alkyl hydrophobic interactions (Figure 10e). Similarly, *S*-**A2** formed one hydrogen bond, involving the H atom of the –NH_2_ group and the O atom from the monomer residue and four π–alkyl hydrophobic interactions (Figure 10f). As observed for the **_T1_**MIP**1** system, **A3** and **A4** interacted with the monomer residues in a similar manner in the **_T1_**MIP**4** system. Both analytes formed two hydrogen bonds between the H atoms of the –NH_2_ group and the O atoms from the monomer residues, one non-classical hydrogen bond and two (**A3**) or four (**A4**) hydrophobic π–alkyl (**A3**) or alkyl–alkyl (**A4**) interactions (Figure 10g and Figure 10h, respectively).

In the **_T1_**MIP**4** models, all analytes interacted with the monomers through hydrogen bonds involving polar –OH or –NH_2_ groups. Hydrophobic interactions were also observed in all cases. Although the total number of interactions between the analytes and the cavity in the **_T1_**MIP**4** system was comparable to or higher than in **_T1_**MIP**1**, the corresponding *ΔE_B_* values were higher, and the adsorption capacities were lower. This may suggest that, in polymers constructed from acidic monomers, acid–base interactions play an important role. In such cases, ionic species could be formed, and the monomer residue and the analyte may associate through strong electrostatic forces. These types of interactions can contribute to the higher adsorption capacities observed for MIPs based on acidic monomers. However, they are typically non-directional and non-specific, which limits their contribution to selective adsorption [52]. This interpretation is consistent with the results of the selectivity tests. While MIPs prepared with acidic monomers exhibited higher adsorption capacities, the highest specificity was observed for polymers based on non-acidic monomers. Acid–base interactions are likely to occur in these systems, as analytes **A1**–**A4** contain a basic –NH_2_ group. Furthermore, differences in charge distribution among the analytes may also influence their interactions with the monomers (Figures S1 and S3 in the Supplementary Materials).

### 2.4. Preliminary Study of Quorum-Sensing Inhibition

To assess the capability of MIPs to inhibit QS—indirectly inferred through the modulation of violacein production rather than the direct quantification of AI-2—preliminary analysis in the microbiological environment was carried out. For that purpose, the percentage inhibition of violacein pigment was quantified in treated versus untreated *Chromobacterium violaceum* ATCC 12472 (Equation (5). This allowed for the determination of MQSIC_50_, in this study defined as the lowest concentration at which violacein production was inhibited by ≥50% with reference to the control sample, as well as to determine the concentration that afforded the highest percentage inhibition of violacein pigment [53,54].

Five MIPs, as indicated previously, were selected for testing, viz., **_T1_**MIP**2**, **_T1_**MIP**4**, **_T2_**MIP**4**, **_T1_**_/**T2**_MIP**4** and **_T1_**MIP**6**, together with NIP**2**. The results are presented in Table 3.

From the results obtained, it is evident that all tested polymers exhibited violacein inhibition, with inhibition percentages ranging from 66.0% for NIP**2** to 79.3% for **_T1_**MIP**2**. In most cases, the percentage of inhibition was directly proportional to the polymer concentration used in the study.

As expected, all MIPs exhibited lower MQSIC (minimum quorum-sensing inhibition concentration) values and higher maximum percentage violacein inhibition compared to the NIP, reflecting their enhanced QS inhibition. This can be attributed to their selective adsorption, as the MIPs were designed to imprint templates structurally analogous to AI-2. The selected compounds demonstrated relatively high specificity towards these templates, enhancing their QS inhibitory activity. NIP**2** demonstrated an MQSIC of 5.0 mg∙mL^−1^, higher than all MIPs, indicating a lack of selectivity and weaker binding interactions with the QS molecules. The highest inhibition observed for NIP**2** was 66.0% at 20.0 mg∙mL^−1^, a level that was lower than that of all tested MIPs, thereby reinforcing the enhanced inhibitory efficacy conferred by molecular imprinting, accentuating the importance of molecular imprinting in improving inhibitory performance.

For the MIPs, the highest percentage violacein inhibition (79.3%) was observed for **_T1_**MIP**2** at a concentration of 20 mg∙mL^−1^. **_T1_**MIP**6** demonstrated the most potent QS inhibition, as indicated by the lowest MQSIC value of 0.3 mg∙mL^−1^, suggesting that this formulation requires the smallest concentration to achieve ≥50% inhibition of violacein production. Generally, MIP**4**, prepared from 2-hydroxyethyl methacrylate in the presence of **T1**, **T2** or a combination of **T1**/**T2**, displayed maximum percentage violacein inhibition, ranging from 70% (**_T2_**MIP**4**) to 78.2% (**_T1_**_/**T2**_MIP**4**), as detailed in Table 3, of which the polymers in the series with the best overall potency were **_T1_**MIP**4** (MQSIC: 1.2 mg∙mL^−1^ and 76.8% maximum inhibition at 5.0 mg∙mL^−1^) and **_T1_**_/**T2**_MIP**4** (MQSIC, 0.6 mg∙mL^−1^ and 78.2% maximum inhibition at 5.0 mg·mL^−1^). It can be postulated from these findings that 2-hydroxyethyl methacrylate is the most suitable monomer for generating high-affinity MIPs, likely due to hydroxyl functionality, which enhances hydrogen bonding with polar sites on the template, thus improving pre-polymerization complex stability and imprinting fidelity.

Interestingly, concentrations exceeding those reported for the highest percentage of violacein inhibition did not further enhance the maximum inhibition observed. This could potentially be attributed to the saturation of the QS inhibitory mechanism, where the target signaling pathways become maximally suppressed at a certain threshold concentration—a hypothesized mechanism that warrants further experimental investigation. Beyond this point, additional inhibitor polymer may not contribute to further inhibition due to receptor saturation or potential off-target interactions that may counteract the inhibitory effect.

The positive control vanillin exhibited the lowest MQSIC (0.02 mg∙mL^−1^) and the highest inhibition (94.9% at 1.25 mg∙mL^−1^), demonstrating its strong QS inhibitory activity. Although the MIPs demonstrated lower potency than vanillin in terms of MQSIC and maximal inhibition percentage, they displayed considerable potential as QS inhibitors, with enhanced specificity compared to the NIP.

Overall, the data presented confirms that molecular imprinting enhances the QS inhibitory activity of polymers, with **_T1_**MIP**4** and **_T1_**_/**T2**_MIP**4** emerging as the most effective polymers, as evidenced by their combined favorable MQSIC values (1.2 mg∙mL^−1^ and 0.6 mg∙mL^−1^, respectively) and high maximal inhibition percentages (76.8% and 78.2%, respectively), further corroborated by earlier physicochemical characterization indicating optimal monomer–template interactions. The choice of monomer and molecular imprinting strategy (template choice) plays a critical role in optimizing inhibitory performance, and further structural optimization could improve efficacy towards QS inhibition.

## 3. Materials and Methods

### 3.1. Reagents

The template molecules, (3R,4S)-tetrahydro-3,4-furandiol (**T1**) and (R/S) 2,2-dimethyl-1,3-dioxolane-4-methanol (**T2**), were purchased from Sigma-Aldrich (Steinheim, Germany). The functional monomers 2-methylprop-2-enoic acid (methacrylic acid, **1**), methylidenebutanedioic acid (itaconic acid, **2**), 4-ethenylbenzoic acid (4-vinylbenzoic acid, **3**), 2-hydroxyethyl methacrylate (**4**), oxiran-2-ylmethyl 2-methylprop-2-enoate (glycidyl methacrylate, **5**), N-(2-methylethyl)prop-2-enamide (N-isopropylacrylamide, **6**) and 1-allyl-2-thiourea (**7**) and the cross-linker, ethylene glycol dimethacrylate, were purchased from Sigma-Aldrich (Steinheim, Germany). The compounds tested in the selectivity analyses, (R/S) 2,2-dimethyl-1,3-dioxolane-4-methanamine (**A1**), 2-amine-1-phenylethanol (**A2**), 2-phenylethylamine (**A3**), 2-(1-cyclohexenyl)ethylamine (**A4**) and vanillin were purchased from Sigma Aldrich (Steinheim, Germany). The solvents, methanol, toluene and acetone, were brought from POCh (Gliwice, Poland), and dimethylsulfoxide (DMSO) was delivered by Thermo Fischer (South Africa). The polymerization reaction initiator, 1,1′-azobiscyclohexanecarbonitrile, was from Merck (Darmstadt, Germany). The HPLC gradient-grade acetonitrile, methanol and formic acid were purchased from Merck (Darmstadt, Germany). Ultra-pure water, delivered from a Hydrolab HLP 5 system (Straszyn, Poland), was used to prepare the water solutions. *Chromobacterium violaceum* (ATCC 12472) was purchased from Davies Diagnostics (Johannesburg, South Africa). Oxoid Luria Bertani broth and agar were delivered by Thermo Fischer (Johannesburg, South Africa).

### 3.2. Synthesis of MIPs

For the purpose of optimizing the material composition, bulk thermal radical polymerization was used with the bulk MIPs coded as MIP**1**–MIP**7** and prepared in the presence of selected template molecules: (3R,4S)-tetrahydro-3,4-furandiol (**T1**, coded as **_T1_**MIP) or (R/S) 2,2-dimethyl-1,3-dioxolane-4-methanol (**T2**, coded as **_T2_**MIP) or an equimolar mixture of **T1** and **T2** (coded as **_T1_**_/**T2**_MIP). Non-imprinted polymers, NIP**1**–NIP**7**, were prepared under the same polymerization conditions but without the template molecule(s) and were treated in the same way as the corresponding MIPs. The experimental amounts of the reagents (moles and masses) used for the preparation of the different types of polymers are listed in Table S1 in the Supplementary Materials. Briefly, the template(s), the appropriate functional monomer and the ethylene glycol dimethacrylate were dissolved in a mixture of toluene and methanol (1:1 *v*/*v*) in a polypropylene tube. The molar ratio of the template to the functional monomer and the cross-linker was set at 1:4:20 (or 0.5:0.5:4:20 for the equimolar mixture of **T1** and **T2**). Finally, the polymerization initiator was added. The homogeneous solutions were purged with nitrogen for 5 min. before sealing. Subsequently, polymerization was carried out in a nitrogen atmosphere for 24 h at 96 °C. The bulk rigid polymers were ground and wet-sieved into particles below 45 μm in diameter. Fine particles were separated by repeated decantation from acetone. The templates were removed from the polymers, using continuous extraction in a Soxhlet apparatus (24–36 h, 80 mL, methanol), followed by an extensive washing with acetonitrile. Elution was monitored by liquid chromatography coupled with LC-MS, and no template residues were detected in the final extracts. The particles were then dried in a vacuum at room temperature.

### 3.3. Characterization Instruments and Techniques

Instrumental analysis was performed using an Agilent 1260 Infinity System (Agilent Technologies, Santa Clara, CA, USA), equipped with a degasser, an autosampler and a binary pump, coupled to a QTRAP 4000 hybrid triple quadrupole/linear ion trap mass spectrometer (AB Sciex, Framingham, MA, USA). The turbo ion spray source was operated in positive mode. The curtain gas, ion source gas 1 and ion source gas 2 were set at 241 kPa, 414 kPa, 276 kPa and “high” instrument units (4.6 × 10^−5^ Torr), respectively. The ion spray voltage and source temperature were 4500 V and 600 °C, respectively. The target compounds were analyzed using Multiple Reaction Monitoring (MRM) mode with the transition *m/z* 133 > 74 (DP = 21 V, CE = 11 V, CXP = 4 V) for **T2**, *m/z* 132 *>* 74 (DP = 51 V, CE = 15 V, CXP = 4 V) for **A1**, *m/z* 138 > 120 (DP = 21 V, CE = 7 V, CXP = 10 V) for **A2**, *m/z* 122 > 105 (DP = 46 V, CE = 15 V, CXP = 6 V) for **A3** and *m/z* 126 > 109 (DP = 41 V, CE = 15 V, CXP = 6 V) for **A4**. **T1** was quantified using a pseudo-MRM transition (*m/z* 105 > 105 (DP = 21 V).

Chromatographic separation was achieved with a Bionacom BionaCore C18 UFPLC Column (100 mm × 4.6 mm, 2.7 µm) from Bionacom (Coventry, UK). The column was maintained at 40 °C at a flow rate of 0.5 mL·min^−1^. The mobile phases consisted of water with 0.2% formic acid as eluent A and acetonitrile with 0.2% formic acid as eluent B. The gradient (%B) was as follows: 0 min 20%, 1 min 20%, 4 min 90% and 7 min 90%. The re-equilibration of the column to the initial conditions lasted for 3 min. The injection volume was 5 µL.

The surface morphology analysis, using scanning electron microscopy (SEM) with a Merlin FE-SEM (Zeiss, Jena, Germany), was performed. The selected samples were Au/Pd sputter-coated prior to analysis. The porosity data for selected samples were determined using the adsorption isotherm of N_2_ at 77 K (BET) on an ASAP 2420 system (Micromeritics Inc., Norcross, GA, USA). The infra-red (FT-IR) spectra were recorded on a Nicolet iS50 FT-IR (Thermo Fisher Scientific, Waltham, MA, USA).

### 3.4. Methods

#### 3.4.1. Dynamic Binding Studies: Template Adsorptivity of MIPs and NIPs

Dynamic binding experiments were performed to evaluate the adsorptivity of the MIPs and NIPs towards **T1** and **T2**. For this purpose, 3 mL empty glass SPE cartridges were filled with 50 mg of **_T1_**MIP**1**–**_T1_**MIP**7**, **_T2_**MIP**1**–**_T2_**MIP**7**, **_T1_**_/**T2**_MIP**1**–**_T1_**_/**T2**_MIP**7** or NIP**1**–NIP**7** particles and secured by glass-fiber frits. To each cartridge, a volume of 1 mL of methanol–water (85:15 *v*/*v*) standard solution of **T1** or **T2** adjusted to pH 3.5 or pH 8.5 was added (for **T1**, the concentration was 3.5 µg L^−1^, and for **T2**, the concentration was 0.5 μg L^−1^). The solution that passed the sorbent was subjected to LC-MS analysis to determine **T1** and **T2’s** remaining concentration. The adsorption capacity of polymers towards **T1** or **T2** was calculated by subtracting the non-adsorbed amount from the initial amount. All measurements were carried out in triplicate. Based on the adsorption measurements, the parameters characterizing the polymers were calculated. The adsorption capacities (*B*, μg∙g^−1^) of MIPs and NIPs were calculated according to Equation (1):

(1) $$B=\frac{(ci\;-\;cf)V}{m}$$

where *V* represents the volume of the solution (L), *c_i_* represents the initial solution concentration (μg∙L^−1^), *c_f_* represents the solution concentration after adsorption (μg∙L^−1^), and *m* is the mass of particles (g). The imprinting factors (IFs) were calculated according to Equation (2):

(2) $$IF=\frac{{B\;}_{MIP}}{{B\;}_{NIP}}$$

#### 3.4.2. DFT-Based Molecular Modeling for Acquisition of Geometrical and ESP-Derived Atomic Partial Charges

The molecular modeling methodology followed a previously established protocol [55]. In brief, the optimized geometries and electrostatic potential (ESP)-derived atomic partial charges of all compounds were obtained using density functional theory (DFT) with the B3LYP/6-311+ G(d,p) hybrid functional, as implemented in Gaussian 16 [56,57]. Initial random configurations of the molecular systems were generated using Packmol [58]. Molecular mechanics (MM) simulations were conducted using the CHARMM force field [59] within the BIOVIA Discovery Studio 2024 environment [60]. Each system underwent two-step energy minimization: first, 100 steps using the steepest descent algorithm, followed by 10,000 steps using the conjugate gradient method. Minimization continued until the root-mean-square (RMS) gradient fell below 0.01 kcal∙mol^−1^∙Å^−1^. Van der Walls’ molecular volumes and surface areas were calculated using BIOVIA Discovery Studio 2024.

The simulations aimed to investigate interactions between the template molecules and functional monomers in the pre-polymerization mixture, thereby identifying the key types of interactions responsible for molecular recognition. Simplified models of pre-polymerization complexes were constructed using template molecules (**T1**, *R*-**T2** or *S*-**T2**) and functional monomers (**1**)–(**7**). For each system, Packmol was used to arrange one template molecule, with four monomer molecules in a simulation box, followed by MM energy minimization to generate the pre-polymerization complex models (coded as **_T1_**PC**1**–PC**7**, *_R_*_-**T2**_PC**1**–PC**7** and *_S_*_-**T2**_PC**1**–PC**7**). Complexation energies (*ΔE_C_*, kcal mol^−1^) were calculated using the following Equation (3):

*ΔE*_C_ = *E*_*system*_ − *E*_*template*_ − 4*E*_*monomer*_, (3)

where *E_system_* is the potential energy of the optimized complex, *E_template_* is the energy of the isolated template, and *E_monomer_* is the energy of a single monomer.

Based on the **_T1_**PC**1** and **_T1_**PC**4** complex models, simplified representations of the polymer cavities were constructed by replacing the double bonds of the monomer molecules with single bonds (by adding hydrogen atoms to the respective carbon atoms) to approximate the C–C bond formation that occurs during polymerization and then removing the **T1** molecule from the complex to obtain the model cavity for further analysis. The analytes *R*- and *S*-**T2**, *R*- and *S*-**A1**, *R*- and *S*-**A2**, and **A3** and **A4** were individually inserted into the model cavity, and their binding energies (*ΔE_B_*) and intermolecular interactions were analyzed. During geometry optimization, constraints were applied to the vinyl C atoms of the monomers (force constants of 10 kcal·mol^−1^·Ǻ^−2^) to preserve the 3D structure of the cavity. The analytes were left unconstrained, mimicking the adsorption step. Adsorption was analyzed using the MM method with the same parameters, as described for the PC model simulations. *ΔE_B_* values (kcal·mol^−1^), between the analytes and the polymer matrix were calculated according to Equation (4):

*ΔE*_*B*_ = *E_system_* − *E_analyte_* − *E*_*cavity*_ (4)

where *E_analyte_* is the energy of the isolated analyte, *E_cavity_* is the energy of the polymer cavity without the analyte, and *E_system_* is the energy of the analyte–cavity system.

#### 3.4.3. Selectivity Testing

Dynamic binding experiments were performed to evaluate the selectivity of MIPs towards **A1**–**A4** and **T2**. For this purpose, 3 mL empty glass SPE cartridges were filled with 50 mg of **_T1_**MIP**1**–**_T1_**MIP**4**, **_T1_**MIP**6**, **_T2_**MIP**1**–**_T2_**MIP**4**, **_T2_**MIP**6**, **_T1_**_/**T2**_MIP**1**–**_T1_**_/**T2**_MIP**4** and **_T1_**_/**T2**_MIP**6** particles and secured by glass-fiber frits. To each cartridge, a volume of 20 mL of methanol–water (85:15 *v*/*v*) standard solution of a mixture of **A1**–**A4** and **T2** was applied (concentration of each was equal to 250 ng∙L^−1^). The solution that passed the sorbent was subjected to LC-MS analysis to determine **A1**–**A4** and **T2’s** remaining concentration. The adsorption capacity of MIPs was calculated by subtracting the non-adsorbed amount from the initial amount according to Equation (1). All measurements were carried out in triplicate.

#### 3.4.4. Inoculation of Bacterial Strains, Media and Culture Conditions

The growth medium chosen for the inoculation of *C. violaceum* ATCC 12472 was Luria Bertani Broth (LBB) and Agar (LBA) (Oxoid), prepared per the manufacturer’s instructions. For preparation of the broth, 25 g of the powder was added to 1 L of sterile water and autoclaved for 20 min at 121 °C. LBA was produced by adding 25 g of Luria Bertani powder and 15 g of bacteriological agar to 1 L of sterile water, which was autoclaved for 20 min. at 121 °C. After sterilization, all media were left overnight at room temperature (25 °C ± 24 h) to confirm sterility before further use. The sterility of the media was confirmed by the absence of turbidity. To prepare the culture, *Chromobacterium violaceum* ATCC 12472 was incubated under aerobic conditions in LBB at 30 °C for 48 h in an orbital shaker incubator (Labcon, Petaluma, CA, USA) at 140 rpm. Inoculum from the broth culture was then streaked out onto an LBA plate containing LBA and incubated at 30 °C for 24 h. After incubation, the purity of the cultures was confirmed by the pure colonies obtained from the streak plate. Thereafter, a single colony from this streak plate was inoculated into a test tube containing 10 mL of LBB and incubated at 30 °C for 24 h at 140 rpm. After incubation, this culture contained approximately 5 × 10^6^ Colony-Forming Units (CFU). This suspension was then diluted by a factor of 10 using LBB as a solvent to achieve a culture suspension of 5 × 10^5^ CFU mL^−1^, which was used for the anti-quorum-sensing assay.

#### 3.4.5. Quantitative Assessment of Violacein Inhibition of MIPs and NIPs

##### Broth Macrodilution Assay

The percentage inhibition of violacein pigment was quantified in treated versus untreated *Chromobacterium violaceum* ATCC 12472, as previously described by Ahmad and co-workers [24] with slight modification. Using aseptic manipulation, a series of concentrations, 20.0, 10.0, 5.0, 2.5, 1.25, 0.625 and 0.3125 mg∙mL^−1^, of **_T1_**MIP**2**, **_T1_**MIP**4**, **_T1_**MIP**6**, **_T2_**MIP**4**, **_T1_**_/**T2**_MIP**4** and NIP**2** were made up in test tubes containing 5 mL of LB broth. This was performed to evaluate the ability of the compound to inhibit violacein production. A 100 μL suspension of *C. violaceum* ATCC 12472 was then added to each tube. Acetone was prepared at 5 mg∙mL^−1^ and added as a negative control for each experiment to ascertain whether the solvent had any antimicrobial properties. Vanillin was prepared at a stock concentration of 5 mg∙mL^−1^ and was used as a positive control (to confirm the susceptibility of the micro-organism to inhibition of quorum-sensing activity). The concentrations used for vanillin were as follows: 0.01, 0.02, 0.04, 0.08, 0.16, 0.31, 0.63 and 1.25 mg∙mL^−1^. A test tube containing untreated *C. violaceum* ATCC 12472 was added as a culture control to ensure the ability of the broth to support growth and as a reference point in the determination of percentage violacein inhibition. The test tubes were then incubated at 30 °C for 48 h with agitation at 140 rpm. Anti-quorum-sensing activity was visually interpreted based on growth and pigmentation (purple). The presence of bacterial growth (turbid) and no color was interpreted as the minimum QS inhibition concentration (MQSIC). The presence of both growth and pigment was interpreted as no anti-quorum-sensing activity and no antimicrobial activity. The results were recorded.

##### Violacein Detection

After incubation, a volume of 1 mL of aliquots from each test tube was transferred into a 15 mL centrifuge tube. The tubes were then centrifuged at 5000 rpm for 10 min to pellet the bacteria containing violacein in an Eppendorf centrifuge 5804 (Merck Millipore). The supernatant was discarded. The remaining bacterial pellet was re-suspended in 1 mL of DMSO (100% DMSO solution), and each centrifuge tube was vortexed (±20 s) until the pellet was solubilized. This was performed to help dissolve the violacein. After adding DMSO and vortexing, the tubes were further centrifuged at 5000 rpm for 7 min. This was performed to separate bacterial cell debris from the solution. After centrifugation, 200 μL of supernatant from each centrifuge tube was placed into two wells of a 96-well microtiter plate. The test was undertaken in duplicate to ensure accuracy. The resultant supernatant was used to determine the violacein content by recording the absorbance at OD595 nm, using a FiterMax F5 multi-mode microplate reader (Molecular Devices, San Jose, CA, USA). The percentage violacein inhibition was calculated using the following Equation (5) [53]:

(5) $$\%\;of\;violacein\;inhibition\;=\frac{culture\;control\;OD595\;nm-test\;OD595\;nm}{culture\;control\;OD595\;nm}\times 100$$

In our study, MQSIC_50_ was defined as the lowest concentration at which violacein production was inhibited by ≥50% relative to the control [54]. This quantitative approach was employed to mitigate the potential variability and subjectivity associated with visual pigmentation assessment, which may differ among researchers [60].

## 4. Conclusions

This study successfully demonstrates the application of molecularly imprinted polymers for quorum sensing inhibition, specifically targeting autoinducer-2 analogs. Characterization studies confirm the successful imprinting of mono-template and dual-template molecules, while binding studies highlight generally higher template affinity (up to 3.36-fold higher affinity) of MIPs compared to NIPs.

Preliminary microbiological evaluation reveals that the synthesized MIPs effectively inhibited violacein production in *Chromobacterium violaceum*, suggesting quorum sensing inhibition, with **_T1_**MIP**4** and **_T1_**_/**T2**_MIP**4** proving to be the most effective polymers. These findings demonstrate the potential of MIPs as quorum sensing inhibitors, which could serve as an alternative strategy to conventional antimicrobial treatments.

Future research will focus on optimizing the MIP synthesis process to improve inhibition efficiency to potentially exceed 80% and broaden its application to other quorum sensing molecules. Additionally, comprehensive cytotoxicity evaluations will be considered to assess the safety of these polymers for potential biomedical applications. In-depth in vivo studies will also be conducted to validate the anti-infective and antibiofilm capabilities of these MIPs. This study provides a foundation for the development of MIP-based strategies in antimicrobial research and quorum sensing modulation.

## Figures and Tables

**Table 1:** Values of complexation energy, *ΔE_C_*, for PC**1**–PC**7** pre-polymerization complex models formed between **T1**, *R*-**T2** or *S*-**T2** and functional monomers (**1**–**7**).

Polymerization Complex Model Code	*ΔE_C_* (kcal∙mol^–1^)		
	T1	*R*-T2	*S*-T2
PC**1**	−60.11	−57.78	−53.21
PC**2**	−135.53	−121.08	−112.82
PC**3**	−73.64	−62.04	−60.74
PC**4**	−39.73	−43.80	−45.43
PC**5**	−46.75	−32.81	−40.25
PC**6**	−41.41	−45.85	−47.58
PC**7**	−44.65	−47.51	−35.68

**Table 2:** Binding energies, *ΔE_B_* and adsorption capacities, *B*, of **A1**–**A4** and **T2** on selected **_T1_**MIP**1** and **_T1_**MIP**4** systems.

Analyte	_T1_MIP**1**		_T1_MIP**4**	
	*ΔE_B_* (kcal·mol^−1^)	*B* (µg·g^−1^)	*ΔE_B_* (kcal·mol^−1^)	*B* (µg·g^−1^)
*R*-**T2**	−14.64	4.35 ± 0.55	−11.67	1.69 ± 0.21
*S*-**T2**	−12.96		−10.57	
*R*-**A1**	−11.97	3.97 ± 0.42	−12.88	0.485 ± 0.051
*S*-**A1**	−13.50		−8.54	
*R*-**A2**	−18.39	9.10 ± 0.54	−10.31	0.697 ± 0.042
*S*-**A2**	−21.27		−8.59	
**A3**	−19.73	9.52 ± 0.69	−6.30	0.485 ± 0.035
**A4**	−21.92	8.75 ± 1.26	−11.03	1.08 ± 0.16

**Table 3:** *Chromobacterium violaceum* QS inhibition data.

Polymer	MQSIC_50_ (mg∙mL^−1^)	MQSIC_50_ Inhibition (%)	Highest Inhibition Conc. (mg·mL^−1^)	Highest Inhibition (%)
**_T1_**MIP**2**	0.6	50.9	20.0	79.3
**_T1_**MIP**4**	1.2	56.5	5.0	76.8
**_T1_**MIP**6**	0.3	67.8	10.0	68.9
**_T2_**MIP**4**	1.2	55.5	20.0	70.0
**_T1_**_/**T2**_MIP**4**	0.6	56.9	5.0	78.2
NIP**2**	5.0	62.5	20.0	66.0
Vanillin (positive control)	0.02	78.3	1.25	94.9

## Data Availability

Data will be made available upon request.

## Supplementary Materials

The following supporting information can be downloaded at: https://www.mdpi.com/article/10.3390/ijms26168015/s1.

## Abbreviations

The following abbreviations are used in this manuscript:

MIP	Molecularly imprinted polymer
QS	Quorum-sensing
AHL	N-acyl-homoserine lactone
AI-2	Autoinducer-2
Lsr	luxS-regulated
NIP	Non-imprinted polymer
dt-MIP	double-templated MIP
st-MIP	single-templated MIP
T1	Template 1, (3R,4S)-Tetrahydro-3,4-furandiol
T2	Template 2, (R/S) 2,2-Dimethyl-1,3-dioxolane-4-methanol
DPD	(S)-4,5-Dihydroxy-2,3-pentanedione
IF	Imprinting factor
PC	Pre-polymerization complex model
ΔEC	Complexation energy
B	Adsorption capacity
FE-SEM	Field emission scanning electron microscopy
BET	Brunauer–Emmett–Teller
BJH	Barrett–Joyner–Halenda
FT-IR	Fourier transform infrared spectroscopy
A1	Analyte 1, (R/S) 2,2-dimethyl-1,3-dioxolane-4-methanamine
A2	Analyte 2, 2-amine-1-phenylethanol
A3	Analyte 3, 2-phenylethylamine
A4	Analyte 4, 2-(1-cyclohexenyl)ethylamine
MQSIC	Minimum quorum sensing inhibition concentration
DMSO	Dimethylsulfoxide
MRM	Multiple reaction monitoring
ESP	Electrostatic potential
DFT	Density functional theory
MM	Molecular mechanics
RMS	Root-mean-square
LBB	Luria Bertani Broth
LBA	Luria Bertani Agar
CFU	Colony-forming units
