# Supplementary material for: A Dual-Template Molecularly Imprinted Polymer to Inhibit Quorum Sensing Molecules: Theoretical Design, Optimized Synthesis, Physicochemical Characterization and Preliminary Microbiological Analysis
Source: Int J Mol Sci. 2025 Aug 19;26(16):8015. doi: 10.3390/ijms26168015 (PMC12386585; doi:10.3390/ijms26168015)
Supplement: Supplementary file 1 [file ijms-26-08015-s001.zip › ijms-3770902-supplementary.pdf]

Supplementary Materials

A Dual-Template Molecularly Imprinted Polymer to Inhibit Quorum Sensing Molecules: Theoretical Design, Optimized Synthesis, Physicochemical Characterization and Preliminary Microbiological Analysis

Khonzisizwe Somandi[1,2], Tama S. Mwale[1,2], Monika Sobiech[3], Dorota Klejn[3], Gillian D. Mahumane[1,2], Joanna Giebułtowicz[4], Sandy van Vuuren[5], Yahya E. Choonara[1,5*] and Piotr Luliński[3*]

[1]Wits Advanced Drug Delivery Platform Research Unit, 7 York Road, Parktown, Johannesburg 2193, South Africa

[2]Department of Pharmacy and Pharmacology, School of Therapeutic Science, Faculty of Health Sciences, University of the Witwatersrand, 7 York Road, Parktown, Johannesburg 2193, South Africa

[3]Department of Organic and Physical Chemistry, Faculty of Pharmacy, Medical University of Warsaw, Banacha 1, 02-097 Warsaw, Poland

[4]Department of Drug Chemistry, Pharmaceutical and Biomedical Analysis. Faculty of Pharmacy, Medical University of Warsaw, Banacha 1, 02-097 Warsaw, Poland

[5]Department of Pharmacy and Pharmacology, Faculty of Health Sciences, University of the Witwatersrand, 7 York Road, Parktown, Johannesburg 2193, South Africa

* Corresponding author: piotr.lulinski@wum.edu.pl

* Co-corresponding author: yahya.choonara@wits.ac.za

Content:

Table S1. Amounts of templates and functional monomers used in the polymerization of 754 μL (4 mmol) of ethylene glycol dimethacrylate as the cross-linker in the presence of mixture of porogens (0.62 μL of toluene and 0.62 μL of methanol) and 15 mg (0.061 mmol, 0.050 $mol \cdot L^{-1}$) of 1,1′-azobiscyclohexanecarbonitrile as the initiator.

Table S2. The *B* values with standard deviations (S.D.) and imprinting factors (IF) for the adsorption of **T1** or **T2** on T1MIP**1** – T1MIP**7**, T2MIP**1** – T2MIP**7**, T1/T2MIP**1** – T1/T2MIP**7** or NIP**1** – NIP**7**.

Table S3. The *B* values of **A1** – **A4** and **T2** on selected MIPs and NIPs.

Figure S1. Molecules of **AI-2**, **T1**, *R*-**T2**, and *S*-**T2** colored according to the partial charge values – negative values are shown as red and positive values as blue.

Figure S2. FT-IR spectra of T1MIP**1**, T2MIP**1**, T1/T2MIP**1** and NIP**1**.

Figure S3. The *R*-**A1**, *S*-**A1**, *R*-**A2**, *S*-**A2**, **A3**, and **A4** colored according to the partial charge values – negative values are shown as red and positive values as blue.

Table S1. Amounts of templates and functional monomers used in the polymerization of 754 μL (4 mmol) of ethylene glycol dimethacrylate as the cross-linker in the presence of mixture of porogens (0.62 μL of toluene and 0.62 μL of methanol) and 15 mg (0.061 mmol, 0.050 mol·L^{-1}) of 1,1′-azobiscyclohexanecarbonitrile as the initiator.

MIPs code	Functional monomer (mg, mmol)	Template **T1** or **T2** or **T1**+**T2** (mg, mmol)
$_{T1}$MIP**1**	methacrylic acid (**1**), 68.9, 0.8	
$_{T1}$MIP**2**	itaconic acid (**2**), 104.1, 0.8	
$_{T1}$MIP**3**	4-vinylbenzoic acid (**3**), 118.5, 0.8	
$_{T1}$MIP**4**	2-hydroxyethyl methacrylate (**4**), 104.0, 0.8	(3R,4S)-tetrahydro-3,4-furan-3,4-diol (**T1**) 20.8, 0.2
$_{T1}$MIP**5**	glycidyl methacrylate (**5**), 113.7, 0.8	
$_{T1}$MIP**6**	N-isopropylacrylamide (**6**), 90.5, 0.8	
$_{T1}$MIP**7**	1-allyl-2-thiourea (**8**), 93.0, 0.8	
$_{T2}$MIP**1**	methacrylic acid (**1**), 68.9, 0.8	
$_{T2}$MIP**2**	itaconic acid (**2**), 104.1, 0.8	
$_{T2}$MIP**3**	4-vinylbenzoic acid (**3**), 118.5, 0.8	
$_{T2}$MIP**4**	2-hydroxyethyl methacrylate (**4**), 104.0, 0.8	(R/S) 2,2-dimethyl-1,3-dioxolane-4-methanol (**T2**) 26.4, 0.2
$_{T2}$MIP**5**	glycidyl methacrylate (**5**), 113.7, 0.8	
$_{T2}$MIP**6**	N-isopropylacrylamide (**6**), 90.5, 0.8	
$_{T2}$MIP**7**	1-allyl-2-thiourea (**8**),	

	93.0, 0.8	
$_{T1/T2}$MIP**1**	methacrylic acid (**1**),<br>68.9, 0.8	
$_{T1/T2}$MIP**2**	itaconic acid (**2**),<br>104.1, 0.8	
$_{T1/T2}$MIP**3**	4-vinylbenzoic acid (**3**),<br>118.5, 0.8	
$_{T1/T2}$MIP**4**	2-hydroxyethyl methacrylate (**4**),<br>104.0, 0.8	**T1** + **T2**
$_{T1/T2}$MIP**5**	glycidyl methacrylate (**5**),<br>113.7, 0.8	10.4 + 13.2 (0.1 + 0.1)
$_{T1/T2}$MIP**6**	N-isopropylacrylamide (**6**),<br>90.5, 0.8	
$_{T1/T2}$MIP**7**	1-allyl-2-thiourea (**8**),<br>93.0, 0.8	

Table S2. The *B* values with standard deviations (S.D.) and imprinting factors (IF) for the adsorption of **T1** or **T2** on $_{T1}$MIP**1** – $_{T1}$MIP**7**, $_{T2}$MIP**1** – $_{T2}$MIP**7**, $_{T1/T2}$MIP**1** – $_{T1/T2}$MIP**7** or NIP**1** – NIP**7**.

Code of polymer	Adsorption of **T1**	
	B (μg g^{-1}) ± S.D.	IF
	pH 3.5	
$_{T1}$MIP**1**	20.9 ± 3.5	1.27
$_{T1}$MIP**2**	30.5 ± 5.1	1.63
$_{T1}$MIP**3**	30.5 ± 5.1	1.86
$_{T1}$MIP**4**	36.3 ± 6.1	1.63
$_{T1}$MIP**5**	16.9 ± 2.9	0.42
$_{T1}$MIP**6**	17.2 ± 2.9	0.78
$_{T1}$MIP**7**	40.7 ± 6.9	1.30
$_{T2}$MIP**1**	20.7 ± 3.5	1.26
$_{T2}$MIP**2**	36.4 ± 6.1	1.94
$_{T2}$MIP**3**	30.5 ± 5.1	1.86
$_{T2}$MIP**4**	34.5 ± 5.8	1.55

${}_{T2}$MIP**5**	16.0 ± 2.7	0.40
${}_{T2}$MIP**6**	17.7 ± 3.0	0.80
${}_{T2}$MIP**7**	27.0 ± 4.6	0.86
${}_{T1/T2}$MIP**1**	24.2 ± 4.1	1.47
${}_{T1/T2}$MIP**2**	36.9 ± 6.2	1.97
${}_{T1/T2}$MIP**3**	29.9 ± 5.0	1.82
${}_{T1/T2}$MIP**4**	18.1 ± 3.1	0.81
${}_{T1/T2}$MIP**5**	23.7 ± 4.0	0.59
${}_{T1/T2}$MIP**6**	19.7 ± 3.3	0.89
${}_{T1/T2}$MIP**7**	15.0 ± 2.5	0.48
NIP**1**	16.5 ± 2.8	-
NIP**2**	18.7 ± 3.2	-
NIP**3**	16.4 ± 2.8	-
NIP**4**	22.3 ± 3.8	-
NIP**5**	40.1 ± 6.8	-
NIP**6**	22.2 ± 3.8	-
NIP**7**	31.4 ± 5.3	-
	pH 8.5	
${}_{T1}$MIP**1**	39.7 ± 2.5	3.36
${}_{T1}$MIP**2**	22.0 ± 1.4	1.74
${}_{T1}$MIP**3**	29.7 ± 1.9	2.70
${}_{T1}$MIP**4**	19.2 ± 1.2	0.97
${}_{T1}$MIP**5**	15.8 ± 1.0	0.75
${}_{T1}$MIP**6**	8.60 ± 0.50	0.38
${}_{T1}$MIP**7**	33.2 ± 2.1	1.14
${}_{T2}$MIP**1**	27.5 ± 1.8	2.33
${}_{T2}$MIP**2**	28.7 ± 1.8	2.28
${}_{T2}$MIP**3**	23.2 ± 1.5	2.10
${}_{T2}$MIP**4**	30.1 ± 1.9	1.52
${}_{T2}$MIP**5**	29.6 ± 1.9	1.42
${}_{T2}$MIP**6**	30.2 ± 1.9	1.31
${}_{T2}$MIP**7**	17.7 ± 1.1	0.61
${}_{T1/T2}$MIP**1**	17.1 ± 1.1	1.44

$_{T1/T2}$MIP**2**	25.5 ± 1.6	2.02
$_{T1/T2}$MIP**3**	31.5 ± 2.0	2.85
$_{T1/T2}$MIP**4**	22.0 ± 1.4	1.12
$_{T1/T2}$MIP**5**	18.6 ± 1.2	0.89
$_{T1/T2}$MIP**6**	22.5 ± 1.4	0.98
$_{T1/T2}$MIP**7**	38.1 ± 2.4	1.31
NIP**1**	11.81 ± 0.80	-
NIP**2**	12.60 ± 0.80	-
NIP**3**	11.00 ± 0.70	-
NIP**4**	19.7 ± 1.3	-
NIP**5**	20.9 ± 1.3	-
NIP**6**	23.0 ± 1.5	-
NIP**7**	29.0 ± 1.9	-
	Adsorption of **T2**	
	B (μg g^{-1}) ± S.D.	IF
	pH 3.5	
$_{T1}$MIP**1**	4.42 ± 0.57	2.21
$_{T1}$MIP**2**	4.30 ± 0.55	1.78
$_{T1}$MIP**3**	3.14 ± 0.40	0.82
$_{T1}$MIP**4**	5.15 ± 0.66	1.71
$_{T1}$MIP**5**	4.24 ± 0.54	0.84
$_{T1}$MIP**6**	4.97 ± 0.64	1.45
$_{T1}$MIP**7**	2.61 ± 0.34	0.78
$_{T2}$MIP**1**	6.26 ± 0.80	3.14
$_{T2}$MIP**2**	5.95 ± 0.76	2.47
$_{T2}$MIP**3**	4.35 ± 0.56	1.13
$_{T2}$MIP**4**	3.08 ± 0.40	1.02
$_{T2}$MIP**5**	3.80 ± 0.49	0.75
$_{T2}$MIP**6**	3.41 ± 0.44	0.99
$_{T2}$MIP**7**	3.46 ± 0.44	1.03
$_{T1/T2}$MIP**1**	4.14 ± 0.53	2.07
$_{T1/T2}$MIP**2**	3.43 ± 0.44	1.42
$_{T1/T2}$MIP**3**	4.47 ± 0.57	1.16

$_{T1/T2}$MIP**4**	3.57 ± 0.46	1.18
$_{T1/T2}$MIP**5**	5.71 ± 0.73	1.13
$_{T1/T2}$MIP**6**	2.46 ± 0.32	0.72
$_{T1/T2}$MIP**7**	3.90 ± 0.50	1.16
NIP**1**	2.00 ± 0.26	-
NIP**2**	2.41 ± 0.31	-
NIP**3**	3.84 ± 0.49	-
NIP**4**	3.01 ± 0.39	-
NIP**5**	5.05 ± 0.65	-
NIP**6**	3.43 ± 0.44	-
NIP**7**	3.36 ± 0.43	-
	pH 8.5	
$_{T1}$MIP**1**	4.72 ± 0.36	1.14
$_{T1}$MIP**2**	4.18 ± 0.32	1.10
$_{T1}$MIP**3**	4.61 ± 0.35	0.96
$_{T1}$MIP**4**	5.91 ± 0.45	1.47
$_{T1}$MIP**5**	4.66 ± 0.35	0.91
$_{T1}$MIP**6**	4.42 ± 0.34	0.64
$_{T1}$MIP**7**	6.93 ± 0.53	1.08
$_{T2}$MIP**1**	6.84 ± 0.52	1.65
$_{T2}$MIP**2**	4.25 ± 0.32	1.12
$_{T2}$MIP**3**	7.11 ± 0.54	1.48
$_{T2}$MIP**4**	5.09 ± 0.39	1.26
$_{T2}$MIP**5**	3.78 ± 0.29	0.74
$_{T2}$MIP**6**	5.35 ± 0.41	0.78
$_{T2}$MIP**7**	3.76 ± 0.29	0.59
$_{T1/T2}$MIP**1**	5.03 ± 0.38	1.21
$_{T1/T2}$MIP**2**	6.46 ± 0.49	1.71
$_{T1/T2}$MIP**3**	5.46 ± 0.41	1.13
$_{T1/T2}$MIP**4**	5.00 ± 0.38	1.24
$_{T1/T2}$MIP**5**	6.29 ± 0.48	1.23
$_{T1/T2}$MIP**6**	7.48 ± 0.57	1.09
$_{T1/T2}$MIP**7**	6.08 ± 0.46	0.95

NIP**1**	4.14 ± 0.31	-
NIP**2**	3.79 ± 0.29	-
NIP**3**	4.81 ± 0.37	-
NIP**4**	4.02 ± 0.31	-
NIP**5**	5.12 ± 0.39	-
NIP**6**	6.89 ± 0.52	-
NIP**7**	6.43 ± 0.49	-

Table S3. Binding capacities of **A1** – **A4** and **T2** on selected MIPs and NIPs.

Code of polymer	B (μg g^{-1})				
	**T2**	**A1**	**A2**	**A3**	**A4**
$_{T1}$MIP**1**	4.35 ± 0.55	3.97 ± 0.42	9.10 ± 0.54	9.52 ± 0.69	8.75 ± 1.26
$_{T1}$MIP**2**	10.0 ± 1.3	9.14 ± 0.97	10.71 ± 0.64	9.51 ± 0.69	8.96 ± 1.29
$_{T1}$MIP**3**	9.0 ± 1.1	9.03 ± 0.96	10.74 ± 0.64	9.52 ± 0.69	8.98 ± 1.30
$_{T1}$MIP**4**	1.69 ± 0.21	0.485 ± 0.051	0.697 ± 0.042	0.485 ± 0.035	1.08 ± 0.16
$_{T1}$MIP**6**	0.676 ± 0.086	0.493 ± 0.052	0.613 ± 0.037	0.427 ± 0.031	0.93 ± 0.14
$_{T2}$MIP**1**	5.42 ± 0.69	4.88 ± 0.52	9.19 ± 0.55	9.46 ± 0.69	8.51 ± 1.23
$_{T2}$MIP**2**	9.7 ± 1.2	9.13 ± 0.97	10.72 ± 0.64	9.52 ± 0.69	8.99 ± 1.30
$_{T2}$MIP**3**	9.9 ± 1.3	8.82 ± 0.94	10.71 ± 0.64	9.52 ± 0.69	9.00 ± 1.30
$_{T2}$MIP**4**	2.21 ± 0.28	0.299 ± 0.032	0.753 ± 0.045	0.675 ± 0.049	0.78 ± 0.11
$_{T2}$MIP**6**	1.20 ± 0.15	0.171 ± 0.010	0.179 ± 0.010	0.147 ± 0.011	0.577 ± 0.083
$_{T1/T2}$MIP**1**	5.97 ± 0.76	5.59 ± 0.59	10.63 ± 0.63	9.51 ± 0.69	8.96 ± 1.29
$_{T1/T2}$MIP**2**	9.9 ± 1.3	9.14 ± 0.97	10.70 ± 0.64	9.52 ± 0.69	9.00 ± 1.30
$_{T1/T2}$MIP**3**	9.8 ± 1.2	9.05 ± 0.96	10.69 ± 0.64	9.52 ± 0.69	8.99 ± 1.30
$_{T1/T2}$MIP**4**	1.61 ± 0.21	1.29 ± 0.14	1.551 ± 0.093	1.48 ± 0.11	1.14 ± 0.17
$_{T1/T2}$MIP**6**	2.16 ± 0.27	1.40 ± 0.15	1.297 ± 0.077	1.297 ± 0.094	1.73 ± 0.25

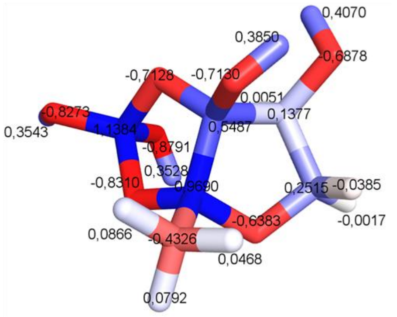

AI-2

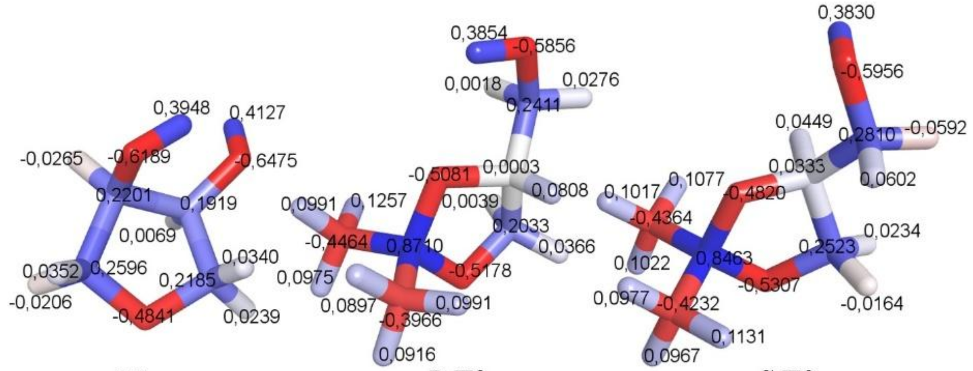

T1 *R*-**T2** *S*-**T2**

Figure S1. Molecules of **AI-2**, **T1**, *R*-**T2**, and *S*-**T2** colored according to the partial charge values – negative values are shown as red and positive values as blue.

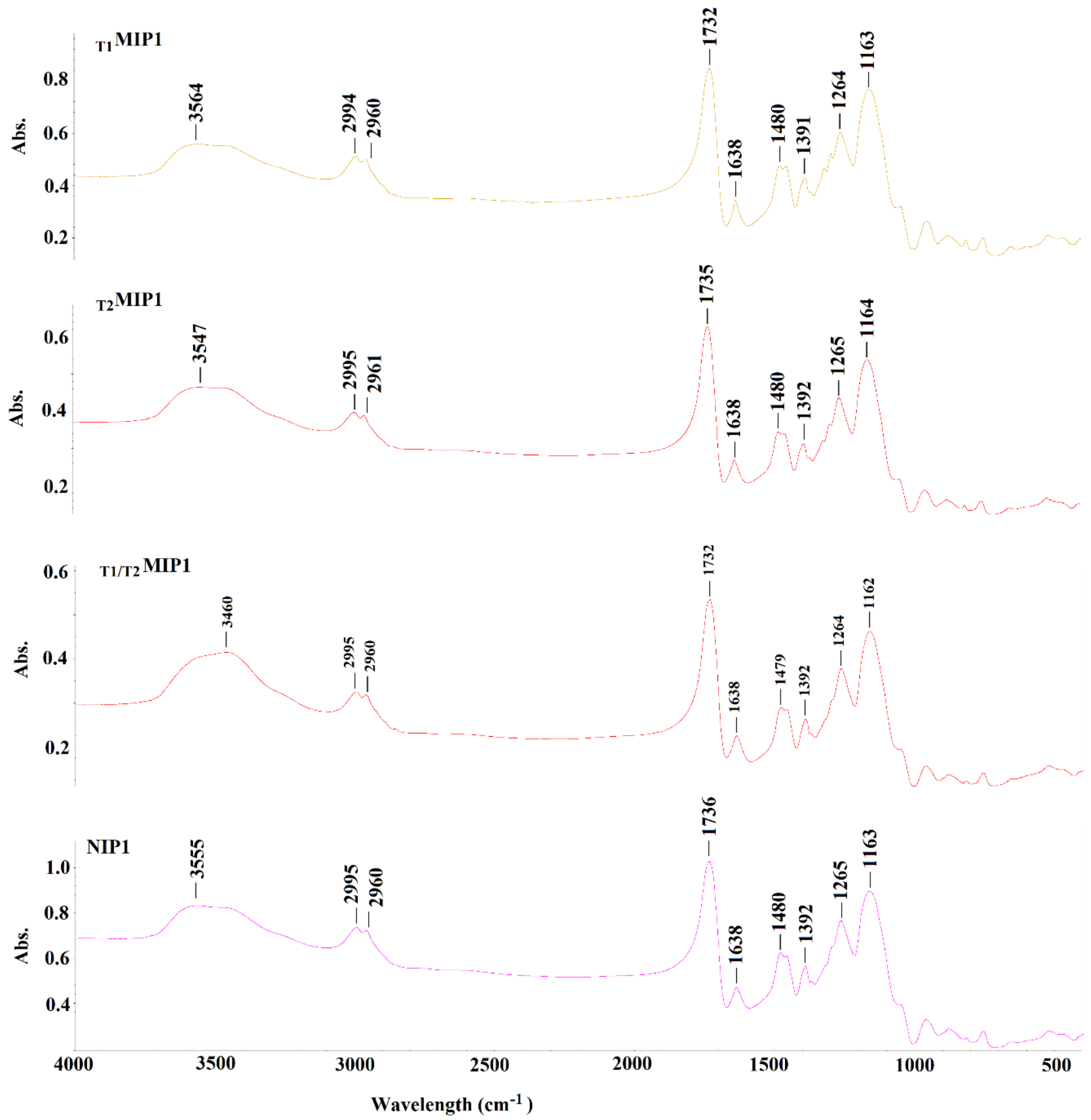

Figure S2. FT-IR spectra of T1MIP**1**, T2MIP**1**, T1/T2MIP**1** and NIP**1**.

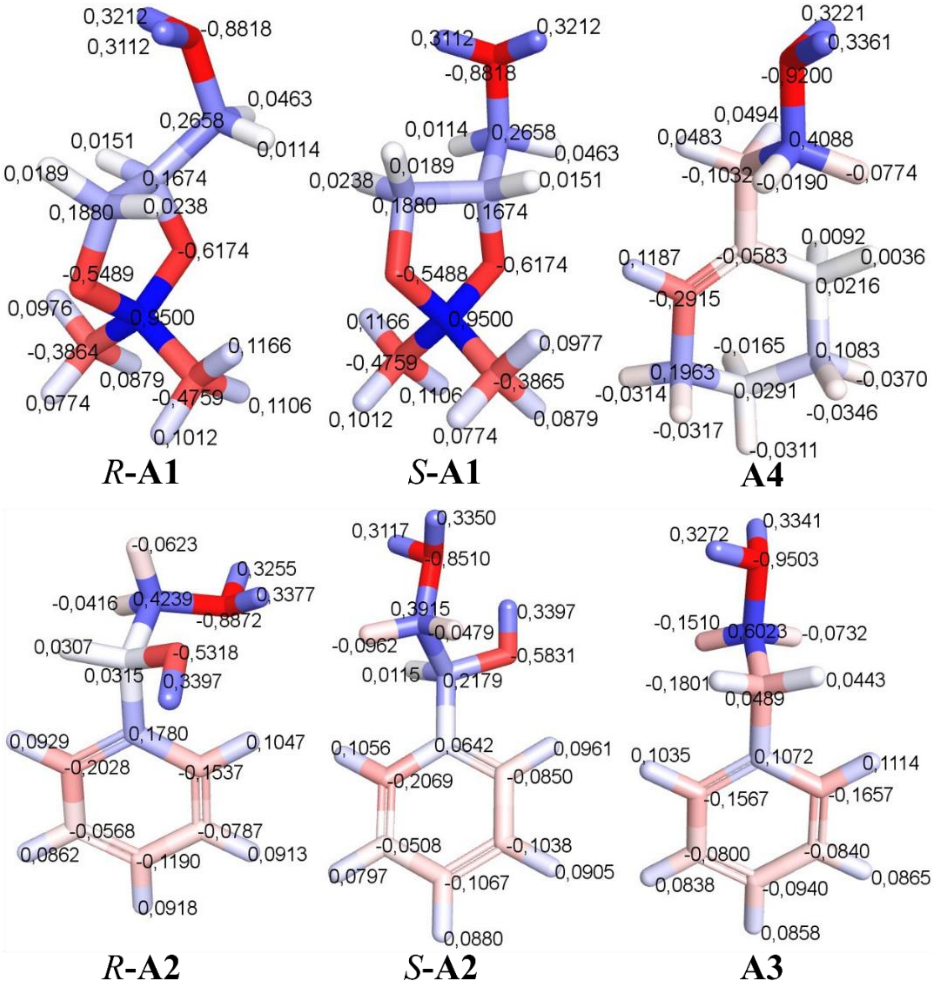

Figure S3. The *R*-**A1**, *S*-**A1**, *R*-**A2**, *S*-**A2**, **A3**, and **A4** colored according to the partial charge values – negative values are shown as red and positive values as blue.
